# Belowground top-down and aboveground bottom-up effects structure multitrophic community relationships in a biodiverse forest

**DOI:** 10.1038/s41598-017-04619-3

**Published:** 2017-06-26

**Authors:** Andreas Schuldt, Helge Bruelheide, François Buscot, Thorsten Assmann, Alexandra Erfmeier, Alexandra-Maria Klein, Keping Ma, Thomas Scholten, Michael Staab, Christian Wirth, Jiayong Zhang, Tesfaye Wubet

**Affiliations:** 1 Leuphana University Lüneburg, Institute of Ecology, Scharnhorststrasse 1, 21335 Lüneburg, Germany; 2 Martin-Luther-University Halle-Wittenberg, Institute of Biology/Geobotany and Botanical Garden, Am Kirchtor 1, 06108 Halle, Germany; 3 UFZ-Helmholtz Centre for Environmental Research, Department of Soil Ecology, Theodor-Lieser-Strasse 4, 06120 Halle (Saale), Germany; 4 German Centre for Integrative Biodiversity Research (iDiv) Halle-Jena-Leipzig, Deutscher Platz 5e, 04103 Leipzig, Germany; 5 University of Kiel, Institute for Ecosystem Research, Olshausenstrasse 75, 24118 Kiel, Germany; 6 University of Freiburg, Institute of Earth and Environmental Sciences, Tennenbacherstr. 4, 79106 Freiburg, Germany; 7 Chinese Academy of Sciences, Institute of Botany, Beijing, 100093 China; 8 University of Tübingen, Department of Geosciences, Soil Science and Geomorphology, Rümelinstrasse 19–23, 72070 Tübingen, Germany; 9 University of Leipzig, Department of Special Botany and Functional Biodiversity, Johannisallee 21–23, 04103 Leipzig, Germany; 10 Zhejiang Normal University, Institute of Ecology, Yinbing Road 688, 321004 Jinhua, China

## Abstract

Ecosystem functioning and human well-being critically depend on numerous species interactions above- and belowground. However, unraveling the structure of multitrophic interaction webs at the ecosystem level is challenging for biodiverse ecosystems. Attempts to identify major relationships between trophic levels usually rely on simplified proxies, such as species diversity. Here, we propose to consider the full information on species composition across trophic levels, using Procrustes correlation and structural equation models. We show that species composition data of a highly diverse subtropical forest―with 5,716 taxa across 25 trophic groups― reveal strong interrelationships among plants, arthropods, and microorganisms, indicating complex multitrophic interactions. We found substantial support for top-down effects of microorganisms belowground, indicating important feedbacks of microbial symbionts, pathogens, and decomposers on plant communities. In contrast, aboveground pathways were characterized by bottom-up control of plants on arthropods, including many non-trophic links. Additional analyses based on diversity patterns revealed much weaker interrelationships. Our study suggests that multitrophic communities in our forest system are structured via top-down effects of belowground biota on plants, which in turn affect aboveground arthropod communities across trophic levels. Moreover, the study shows that the consequences of species loss will be more complex than indicated by studies based solely on diversity.

## Introduction

Interactions of multitrophic communities drive ecosystem functions and the provisioning of ecosystem services^1–4^. Food web analyses have revealed important interaction pathways for subcomponents of these community webs^5, 6^. However, at the ecosystem level such analyses based on direct feeding observations are only feasible for moderately diverse systems with typically tens to a few hundred species^7, 8^. In highly biodiverse regions analyzing community webs of higher eukaryotic species and including data on microorganisms at an ecosystem scale requires alternative approaches^9, 10^. This is because direct observation of the numerous interactions is extremely resource intensive and sometimes hardly feasible for entire communities (e.g. feeding preferences of many predators and herbivores, matter fluxes among belowground microbes). In such cases, covariance-based approaches have proven useful in providing a framework for testing hypotheses on the structuring of community webs^9–12^.

A frequently used approach to unravel such relationships among highly diverse communities at the ecosystem level relies on simplified proxies, such as species diversity or abundance^12, 13^. However, diversity proxies are usually not sufficient to unveil the functional structure of community webs^4, 14, 15^. Finer resolution data on species compositions for a whole-ecosystem-level analysis of community relationships have been used less frequently^9, 16, 17^. This is despite the fact that correlations among species compositional patterns and analyses of subsets of such webs have revealed the usefulness of these data for identifying key interaction pathways^11, 18–21^. Importantly, these webs also include indirect and non-trophic relationships^12, 22^ that necessarily remain undetected by analyses of feeding interactions, but play important roles in structuring ecosystems^9, 23^.

The analysis of such relationships across trophic levels faces a major difficulty since there is a wealth of plant, animal, and microbial taxa, for which we do not even have the vaguest idea about how they interact. As the number of possible relationships among species increases exponentially with species richness, statistical tools are required to distinguish probable from possible interactions among taxa in species-rich systems. Ideally, these tools allow identifying the direction of community relationships across multiple trophic levels, i.e. a discrimination of top-down and bottom-up effects. Powerful tools to provide insight into patterns of community congruence among organism groups (such as Procrustes analysis^24^) and the causal links in ecological communities (such as structural equation models (SEM)^25^) have been developed in the past, but they have been used largely independently from each other^9–12^. Combining such methods to integrate the large information content of multivariate community data sets into statistical modeling has a high potential to advance our understanding of community webs by helping to develop informed hypotheses on the structure and functional role of such webs in complex ecosystems.

The structure of community webs is particularly poorly understood for species-rich ecosystems, such as subtropical and tropical forests, which are strongly affected by species interactions across trophic levels^26^. Herbivores, plant pathogens, and mutualists, such as mycorrhizae, have been shown to influence the composition and diversity of tree communities^27–30^. However, trees are long-lived individuals and the influence of their antagonists and mutualists can change over time^31, 32^ as trees grow larger and exert strong and long-lasting control on biotic (e.g. the availability of specific food resources for specialized consumers) and abiotic (e.g. soil pH, microclimate, structural heterogeneity) characteristics of forest ecosystems. In mature forests, the influence of bottom-up effects of the producer level on higher trophic levels might therefore predominate over potential top-down effects of consumers on trees. Many studies have reported strong bottom-up, tree-controlled impacts on the diversity or composition of individual groups of organisms at higher trophic levels, both belowground^20, 33^ and aboveground^34, 35^. However, whether bottom-up control predominates when it comes to the structuring of community associations across the multiple groups of organisms at multiple trophic levels in biodiverse forests remains poorly explored.

Here, we use species composition data of 25 trophic groups of plants, arthropods, and microorganisms—representing a total of 5,716 species and operational taxonomic units—to unravel key community relationships and their potential bottom-up and top-down effects in a highly diverse forest. The data were collected on 27 study plots that represented the range of successional stages and woody plant diversity typically encountered in the highly diverse study region. We developed an approach that combines the strengths of Procrustes correlation analysis of principal components analyses (PCA) and SEM (using the site scores of the first two PCA axes) to analyze below- and aboveground multitrophic community patterns (see Methods). We explored the potential causal links between trophic levels by testing for direct and indirect relationships and the support for bottom-up and top-down control of these relationships, while accounting for potential environmental covariation. Moreover, we compared the community-based relationships to species richness and diversity relationships among the 25 trophic groups to assess the degree to which relationships among taxa are potentially driven by diversity patterns.

We hypothesized that the main relationships both below- and aboveground are primarily bottom-up controlled, because plants are key drivers of nutrient flows and environmental conditions in many ecosystems^11, 12^. Considering the longevity of trees, they might have a stronger impact on the long-term dynamics of the multitrophic community structure in forests and outweigh the temporally more variable top-down effects of pathogens, herbivores, and soil symbionts. We expected to find a strong discriminative power of species composition data that might complement the more frequently conducted analyses of diversity patterns in biodiverse systems.

## Results

**Figure 1:**
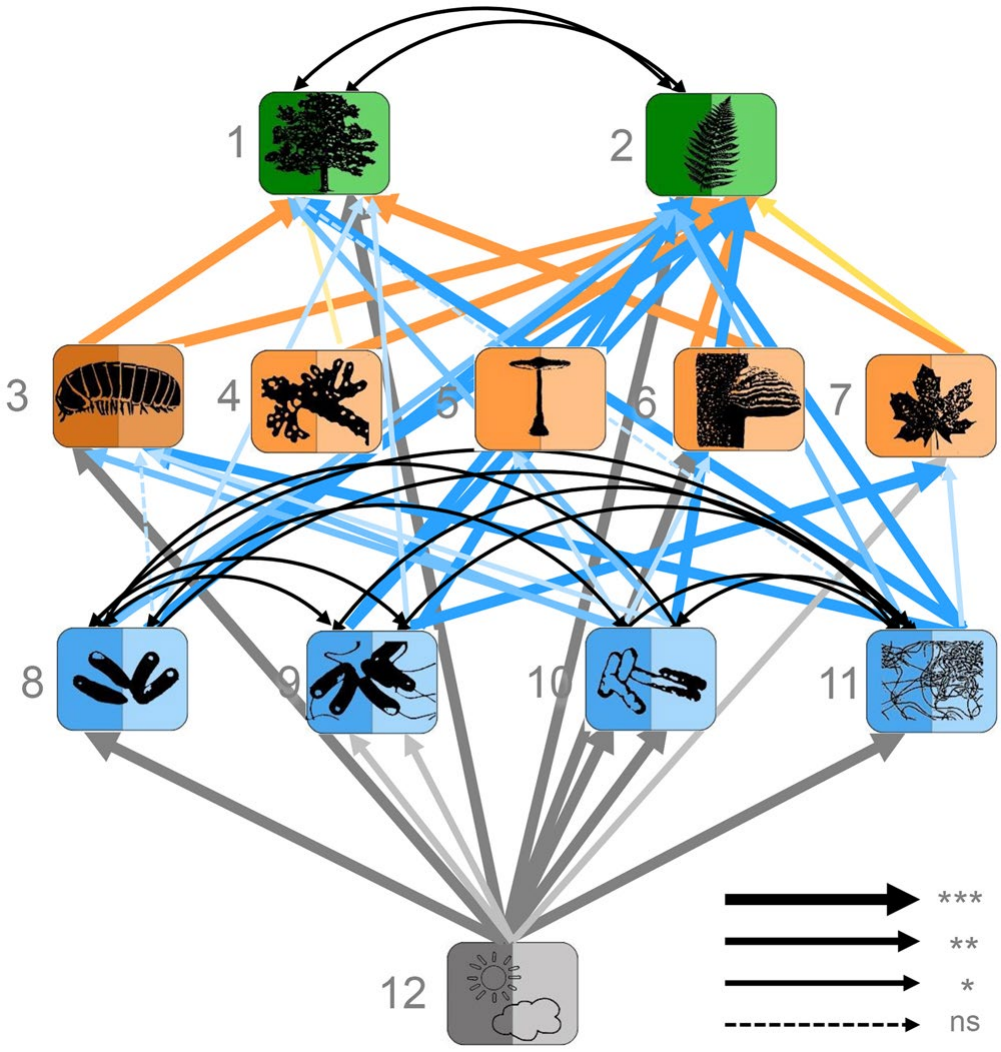
Top-down control in the belowground community web. Structural equation model across trophic levels based on community structure, represented for each organism group by the first two axes of principal components analyses (PC1: darker shade, PC2: lighter shade) on species identities and relative abundances (χ² = 132.4, P = 0.127, DF = 115, RMSEA = 0.075, P-value RMSEA = 0.266, AIC = −1470.4, N = 27). Relationships are controlled for environmental dependencies, scaled proportional to their significance (***P ≤ 0.001; **P ≤ 0.01; *P ≤ 0.05; ns nonsignificant). For clarity, only covariances ≤0.01 are plotted. See Supplementary Table S3 for detailed model ouput with path coefficients and error terms, and Fig. S4 for an alternative presentation. Colors of boxes and corresponding arrows indicate different trophic or functional groups. Groups are: tree layer plants (1), herb layer plants (2), macrofaunal decomposers (3), arbuscular mycorrhizae (4), ectomycorrhizae (5), saprophytic fungi (6), pathogenic fungi (7), Acidobacteria (8), Alphaproteobacteria (9), Bacteriodetes (10), Chloroflexi (11), environment (12).

**Figure 2:**
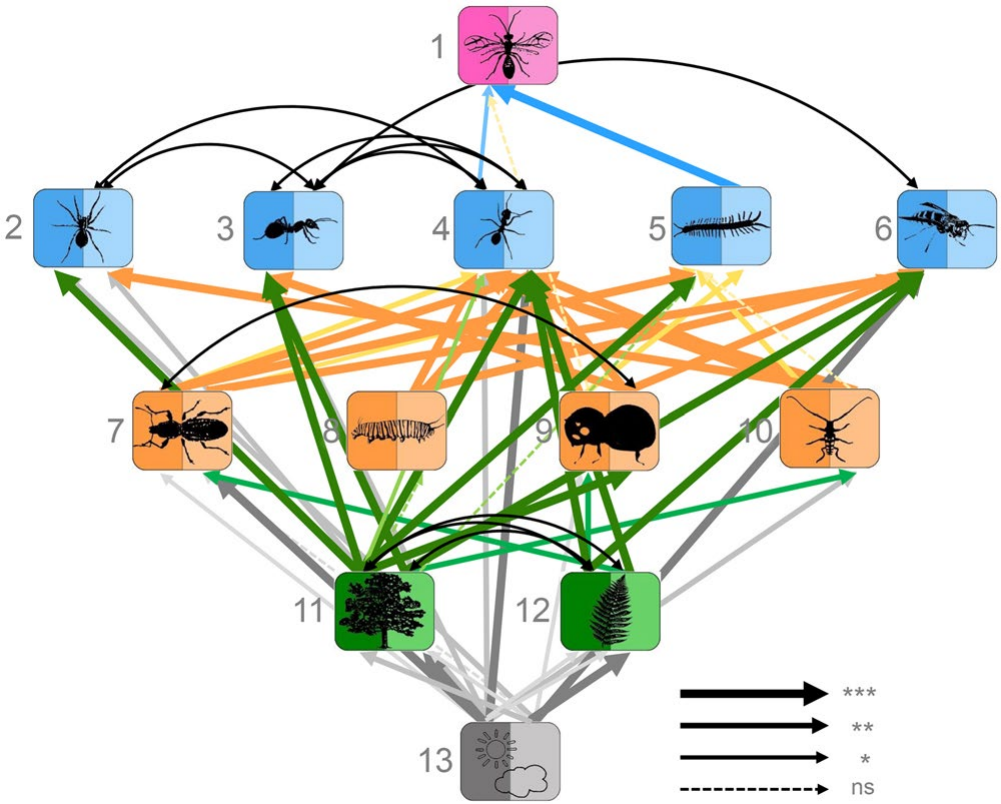
Bottom-up control in the aboveground community web. Structural equation model across trophic levels based on community structure, represented for each organism group by the first two axes of principal components analyses (PC1: darker shade, PC2: lighter shade) on species identities and relative abundances (χ² = 206.9, P = 0.119, DF = 184, RMSEA = 0.068, P-value RMSEA = 0.295, AIC = −353.1, N = 27). Relationships are controlled for environmental dependencies, scaled proportional to their significance (***P ≤ 0.001; **P ≤ 0.01; *P ≤ 0.05; ns nonsignificant). For clarity, only covariances ≤0.01 are plotted. See Supplementary Table S4 for detailed model ouput with path coefficients and error terms, and Fig. S5 for an alternative presentation. Colors of boxes and corresponding arrows indicate different trophic or functional groups. Groups are: Parasitic Hymenoptera (1), spiders (2), omnivorous ants (3), predatory ants (4), centipedes (5), predatory wasps (6), weevils (7), lepidopteran caterpillars (8), bark beetles (9), longhorn beetles (10), tree layer plants (11), herb layer plants (12), environment (13).

Our analyses revealed an unexpectedly high number of significant relationships among the community composition patterns of all trophic groups (123 out of 300 comparisons, i.e. 41%, were significant; Supplementary Table S1. Even when adjusting P-values to the expected number of false discoveries due to multiple testing, 30% of all comparisons remained significant). This was true for both the below- and aboveground data, even after controlling for the influence of abiotic environmental conditions (Figs 1 and 2) which vary with the spatial location or successional age of the plots (in particular those represented by PC1; see Table S2).

For the belowground compartment top-down control received more statistical support (AIC -1470.4 vs. AIC -1452.7 for bottom-up control; Fig. 1, Supplementary Fig. S1), with strong influences of prokaryotic (bacteria) and eukaryotic (fungi and macrofaunal decomposers) organisms on plant species composition (Fig. 1). Bottom-up effects of plant species composition on belowground heterotrophic organisms were comparatively weaker. Strikingly, both ectomycorrhizal and arbuscular mycorrhizal fungi were not significantly related to tree layer woody plants in the bottom-up model. By contrast, arbuscular mycorrhizal fungi appeared to strongly influence plants in the top-down model (Fig. 1). Similarly, we found stronger direct relationships between bacteria (in particular Alphaproteobacteria and Bacteroidetes) and plants in the top down model as compared to the bottom-up model. Results were qualitatively similar when belowground models were fit with an alternative trophic structure where the prokaryotes (bacteria) were considered to be on the same trophic level as the eukaryotes (fungi and macrofaunal decomposers; data not shown).

In contrast to the belowground compartment, aboveground community relationships were clearly determined by bottom-up effects (AIC -353.1 vs. AIC -324.6 for top-down control; Fig. 2, Supplementary Fig. S1). In particular, our analyses revealed strong direct, non-trophic effects of plants on predators, whereas effects on herbivores were less pronounced (Fig. 2). Predator community structure was also strongly influenced by bottom-up effects of herbivores, with strong linkages particularly between predatory ants and herbivores (Fig. 2). Relationships between herbivores and plants were more obvious in the, overall less supported, top-down model (Supplementary Fig. S1).

The analyses of species richness and Shannon diversity patterns among organism groups also suggested a stronger statistical support for top-down than for bottom-up control for the belowground compartment, but this was most obvious for the diversity patterns (whereas the analyses of species richness supported both bottom-up control and top-down control, with ΔAIC < 2; Supplementary Fig. S2). For the aboveground communities, the diversity analyses indicated bottom-up control (Supplementary Fig. S3), concordant with the community models. In contrast to the community patterns, however, the richness and diversity analyses showed weaker connections across trophic levels. For example, the belowground top-down models indicated much fewer and qualitatively different plant-microorganism relationships than those identified in the community data analyses. For instance, there were no significant effects of arbuscular mycorrhizal fungal richness or diversity on tree layer woody plant species richness and diversity (Supplementary Fig. S2). Likewise, significant relationships with tree and herb layer plants were only detectable for predatory groups and longhorn beetles in the most-supported aboveground bottom-up models (Supplementary Fig. S3).

## Discussion

In the face of increasing global environmental change, disentangling the structuring of community relationships is crucial to developing a better understanding of the consequences of biodiversity loss and species compositional changes on ecosystems and their functioning^1, 3^. Our study—based on several thousand taxa of plants, invertebrates, and microorganisms—suggests that top-down effects of belowground plant symbionts, pathogens, and decomposers structure the plant community composition of a biodiverse forest, which in turn shapes aboveground arthropod community composition via bottom-up effects across several trophic levels.

In contrast to the hypothesized bottom-up control, our analyses suggested a strong impact of belowground top-down effects on plant communities in our study system. Although plant communities can strongly shape the habitat conditions for taxa at higher trophic levels^33, 36^, feedback effects of both plant antagonists and mutualists might result in an overall strong top-down control of plant community composition^27, 29^. Our findings are in line with observations in other species-rich forests that have indicated strong effects of soil fungal pathogens, bacteria, and mycorrhizae on plant community composition^28–30^. An important role of top-down effects is indicated by strong links between microorganisms and plants in the top-down model that are not found or less evident in the bottom-up model, such as for Alphaproteobacteria and Bacteroidetes. Alphaproteobacteria are known to interact with plants predominantly as symbionts and pathogens^37^, whereas members of the Bacteroidetes are specialized on carbon mineralization^38^, influencing the recycling of organic matter, nutrient availability, and therefore ultimately plant community composition.

Likewise, strong effects of arbuscular mycorrhizal fungi in the top-down model indicate an important role of these fungi for tree composition, which contributed to the stronger support of top-down control in the belowground compartment of our study system. In this respect, it is striking that in the bottom-up model, neither ectomycorrhizal nor arbuscular mycorrhizal fungi were significantly related to tree layer woody plants. While scale-dependence of the strength of associations among microorganisms and plants might contribute to explaining these results^11, 39, 40^, recent analyses in a tropical forest showed that the strongest correlations between tree and soil microbial community composition occur at a neighborhood scale of 20 m (ref. 11), which is comparable to the plot-level scale of our study. It is more likely that many mycorrhizal fungi are associated with plant species that do not occur in the tree layer of our study plots^41^, which is also reflected by the fact that the only significant bottom-up effect of plants on mycorrhizae was that of herb layer plants on arbuscular mycorrhizae (Supplementary Fig. S1). This potentially makes tree layer plant composition an insufficient indicator of both ectomycorrhizal and arbuscular mycorrhizal community structure, which fits to the non-specific web that mycorrhizal fungi establish among tree species^42, 43^. Top down effects of both types of mycorrhizae on plants indicate that despite the known differences in biodiversity ratios of these two groups of mycobionts and their plant partners and despite their mobilizing of different soil resources^44^, their effects have similar directions in the belowground community web. This suggests that plant nutrition by the fungi and not the provisioning of photosynthates by the plants is the driving force within the bidirectional trophic relationships of mycorrhizal associations.

Additional effects on plant community composition were mediated by saprophytic fungi and macrofaunal decomposers (we note that our study does not include mesofaunal decomposers). Both organism groups contribute to nutrient recycling and may therefore be important for the mobilization of soil resources utilized by plants.

In contrast to the belowground compartment, the strong support for bottom-up control of aboveground biota by plants is in accordance with the hypothesized patterns. Our results conform with previous findings on diversity relationships among individual taxa in our study system as well as in others^9, 16, 34^. These findings emphasize the important role of plant communities in directly structuring community patterns not only of herbivores, but also of predators and parasitoids^35^, in the aboveground forest compartment. In this context, it is notable that our SEM analysis showed comparatively more links between plant and predator communities than between plant and herbivore communities. Previous studies have shown that even higher trophic levels, such as predators, can be strongly influenced by plant community composition^9, 16^, and our results highlight the importance of such, probably largely non-trophic, effects for community patterns at the whole-ecosystem scale. Non-trophic bottom-up effects might occur, for example, through modification of abiotic properties or resource availability. Recent studies have indicated that leaf density, bark structure, litter properties, and other morphological characteristics, which vary among tree species and influence microclimate and habitat space, can significantly affect the species composition of predator assemblages^19, 45^. In some cases, the relationships between plants and predators could even involve a trophic component, because many “predators” might utilize plant resources, such as nectar from extrafloral nectaries or flowers, in addition to animal prey. This is well-known for ants, but also applies to rather strict predators such as many spiders and wasps^46^. However, woody plants with extrafloral nectaries are uncommon at our study site^47^, making a strong impact of such effects less likely. Nevertheless, the results of our study reveal interesting directions for further research on the effects of plant species composition on higher trophic levels.

The strong support for the bottom-up model in the aboveground compartment does not necessarily preclude potential top-down effects from contributing to the structuring of community patterns across trophic levels^9, 27^. Such top-down effects were implied in our study by the links between herbivores and plants in the top-down model. However, these seem to have less effect on the main relationships, as indicated by the poor fit of the top-down model. Rather, our results point to the intriguing hypothesis that the structuring of aboveground arthropod communities is indirectly influenced by the strong top-down effects that belowground microorganisms have on the plant communities. Such indirect structuring effects across ecosystem compartments, mediated by producer communities, have repeatedly been shown for specific taxa or subcomponents of community webs^48–50^, but remain less well explored for the structuring of entire, species-rich ecosystems. While the complexity of our data hinders us from directly testing these indirect effects across compartments, such linkages would have important implications for our understanding of how the effects of biodiversity loss and environmental change might cascade through ecosystem compartments and trophic levels^48, 51^.

It is notable that both tree and herb layer plant community composition showed a large number of links with higher trophic levels (in both the above- and belowground compartments). While this reflects the degree to which taxa are associated with specific vegetation strata, it particularly highlights the important role of the herb layer (which provides the recruits for the next generation of tree species) in the structuring of the community webs in species-rich forests^41, 52^.

The analyses of species richness and diversity patterns yielded fewer and qualitatively different links across trophic levels compared to the community composition analyses. This shows that our approach can provide insight into the structuring of ecological communities that apparently are not revealed by analyses of richness or diversity patterns. The comparatively weak connections across trophic levels in the richness and diversity analyses (especially between plants and herbivores) deviate from the findings of a similarly comprehensive study^12^, which, however, was conducted in a much less complex, experimental grassland system and did not differentiate among groups of herbivores or predators. On the other hand, our results are consistent with those of previous studies on individual trophic groups in our study system, which found that many effects of woody plant species richness or diversity acted on higher trophic level abundance rather than on richness or diversity patterns^34, 53, 54^. Moreover, species richness relationships among plants, arthropods, and microorganisms in our studied forests were found to be highly non-linear across spatial scales^39^. These results underline that addressing taxon-specific responses to the mechanisms underlying the structuring of community relationships at the local scale requires consideration of species identities and distributions. Analyses based on species richness or diversity may therefore strongly benefit from being complemented by analyses of community composition data^9, 55^.

The relationships shown in the community and diversity webs were controlled for changes in environmental conditions among study plots. Nevertheless, strong effects particularly of the first environmental PCA axis (PC1) on many organism groups indicate that the structuring of multitrophic communities of course also depends on abiotic conditions that vary spatially or with succession. The environmental variables particularly associated with PC1 were local temperature patterns and soil nutrient conditions (C and N concentrations). Many previous studies have highlighted the importance of these environmental parameters on the abundance and species composition of above- and belowground taxa^9, 17, 20, 33^. Our study shows that in addition to such environmental effects, interactions among organism groups play an important role in structuring community relationships across trophic levels.

In summary, our results show how detailed community analyses that go beyond simplified diversity metrics can help to reveal important relationships and their relative dependence on top-down and bottom-up control across ecosystem compartments even in highly species-rich ecosystems. Our analytical approach emphasizes the importance of species identities and community composition in revealing many of the trophic and non-trophic linkages that contribute to structuring biodiverse ecosystems, linkages that may go unnoticed with more aggregated diversity data. Our approach allows for the simultaneous incorporation of species-level data, and their potential top-down or bottom-up effects, for the multitude of organism groups that make up ecosystems and their functioning. At the same time, it allows controlling for covariation that might arise from shared environmental influences, which means that our method can help to reveal direct or indirect species interactions. Our findings indicate that top-down control of microbial plant symbionts and pathogens has a strong structuring effect on the plant communities in our biodiverse forest system. These effects might indirectly cascade to the aboveground compartment via bottom-up effects of plants on arthropod communities. Ultimately, an in-depth mechanistic understanding of the observed patterns will benefit from direct observations of interactions among species to validate our correlational approach, but obtaining such data for the full set of thousands of species in biodiverse ecosystems is illusive. Our approach might be modified to help identify key species sets for further experimental and observational research, for example by the sequential filtering of species from the community matrices that contribute significantly to explaining community concordance.

## Methods

### Study site and design

The study was conducted in the Gutianshan National Nature Reserve (GNNR), Zhejiang province, South-East China (29°14′N; 118°07′E). The reserve comprises about 8,000 ha of evergreen mixed broadleaved forest on sloping terrain (300–1,260 m a.s.l.). Dominant tree species are *Schima superba* Gardn. et Champ. and *Castanopsis eyrei* (Champ. ex Benth.) Tutch. Mean annual temperature is 15.3 °C and mean annual precipitation ca. 2000 mm (ref. 56).

In 2008, we established 27 study plots of 30 m × 30 m in the reserve. We used a stratified sampling design to capture the typical range of woody plant species richness (25–68 species per plot) and successional age (from <20 to >80 years since the last logging events) encountered in the GNNR. The study plots were randomly spread across the accessible parts of the reserve^57^.

### Species data

Our analyses were based on 25 groups of plants, arthropods, and microorganisms, with a total of 5,716 species or operational taxonomic units that are characterized, in the case of heterotroph organisms, by specific functional characteristics of feeding ecology and trophic rank^39^.

Plants, as the stand-structuring organisms, were subdivided into woody species forming the tree and shrub layers (all individuals >1 m height^57^), and species forming the herb layer (<1 m plant height^41^).

Microorganisms comprised arbuscular mycorrhizal fungi (AMF), ectomycorrhizal fungi (ECM), saprophytic fungi, parasitic fungi, and the eight most abundant bacterial phyla (which accounted for more than 93% of all sequence reads: Acidobacteria, Actinobacteria, Alphaproteobacteria, Bacteroidetes, Betaproteobacteria, Chloroflexi, Deltaproteobacteria, Gammaproteobacteria).

Arthropods were parasitoids, predators, herbivores/primary consumers, and macrofaunal decomposers. Parasitoids comprised parasitic wasps (Hymenoptera: Braconidae, Chrysididae, Eurytomidae, Ichneumonidae, Leucospidae, Mutillidae, Pompilidae, Trigonalyidae). Predators were spiders (Arachnida: Araneae), centipedes (Chilopoda), cavity-nesting solitary wasps (Hymenoptera: Pompilidae, Sphecidae, Vespidae), and ants (Hymenoptera: Formicidae). Because many ant species are not strict predators, we subdivided the ant data based on the trophic position of the ant genera^54^ into strictly predatory ants and omnivores that, in addition to scavenging and hunting, also feed on plant-based resources such as honeydew. Herbivores/primary consumers (termed herbivores hereafter) comprised moth and butterfly caterpillars (Lepidoptera), weevils (Coleoptera: Curculioninae), longhorn beetles (Coleoptera: Cerambycidae), and bark beetles (Coleoptera: Scolytinae). Macrofaunal decomposers were millipedes (Diplopoda) and isopods (Isopoda) (analyzed together as “decomposers”, see below).

The woody plant communities of each plot were inventoried completely, with all tree and shrub individuals >1 m height, in 2008^57^. At the same time, herb layer plant communities were surveyed with abundance and cover estimates in the central 10 m × 10 m of each plot (<1 m height^41^).

Microorganisms were sampled with soil cores (eight samples per plot in September 2012 from the upper 10 cm of soil, pooled to four composite samples per plot). Soil cores were sieved, cool-transported to the field lab and freeze-dried for molecular analysis. Microbial DNA was extracted from 1 g of each of the composite freeze-dried soil samples using the MoBio soil DNA extraction kit. Fungal and bacterial communities were analyzed by pyrotag amplicon sequencing of the fungal ITS^58^ and the V3-V5 region of the bacterial 16 S rRNA genes^59^. Sequence datasets were further quality filtered, normalized to enable an unbiased comparison among plots to 10,000 fungal ITS and 20,000 bacterial 16 S rDNA reads per plot using MOTHUR^60^. Sequences were clustered into species-level operational taxonomic units (OTUs) using CD-HIT-EST at 97% pairwise similarity threshold^59^. Bacterial 16 S OTU representative sequences were assigned taxonomy against the Silva SSU reference database while fungal ITS OTU representative sequences were classified against the UNITE database^40^. Non-target taxa OTUs as well as singletons, doubletons and tripletons (which have a high probability of originating from artificial sequencing errors^61^) were removed from the dataset. For further details on sample processing and sequencing see ref. 39.

The fungal reference sequences were assigned to ecological functional groups on the basis of sequence similarity using the default parameters of the GAST algorithm^62^ against the functional reference dataset^40^. The bacterial dataset was split into phyla (except for the phylum Proteobacteria, which was further split into subphyla), because the functional grouping of bacterial communities is currently challenging^38^. The community data for most of these phyla were highly correlated (Procrustes correlations *P* < 0.001 and t >0.8 in many cases) and showed similar Procrustes correlation patterns with plants and other below ground taxa. Because this indicates highly redundant information among bacterial groups, we used Acidobacteria (highly correlated with Actinomycetes and Gammaproteobateria and exhibiting similar Procrustes correlation patterns as these two groups across the plants and other below ground taxa), Alphaproteobacteria, Bacerioidetes and Chloroflexi for further analysis. Delta- and Betaproteobacteria were excluded from the analysis, as they showed no significant Procrustes correlations with plants and other belowground taxa. The four retained taxa altogether accounted for more than two-thirds (73%) of the abundance and 67% of the OTUs within the eight most abundant phyla and can therefore be assumed to play a key role in structuring major community relationships of bacteria in the belowground compartment of our study system.

Arthropods were sampled during the main growing seasons of the years 2008–2012^39^. Epigeic spiders, centipedes, epigeic ants, weevils, isopods and diplopods were sampled with pitfall traps (4 traps per plot from March to September 2009^53^). Beating of understory trees and shrubs was used to sample Lepidopteran larvae, arboreal spiders, and ants (25 plant individuals per plot on three sampling dates in 2011 and 2012^34^). Cavity-nesting predatory wasps and the associated parasitic wasps were sampled with reed-filled trap nests (2 traps per plot from September 2011 to October 2012^63^). Longhorn beetles, bark beetles, and canopy ants were sampled with flight interception traps (4 traps per plot from May to August 2010^39^). In addition, ants were sampled with standardized protein and carbohydrate baits (36 baits per plot in May 2012^47^). All arthropods were identified to morphospecies or, where possible, species.

### Environmental data

To account for potential covariation among organism groups due to similarities in the response to general plot characteristics, important spatial and environmental variables were included in our analyses. These variables characterize variation in both above- and belowground conditions that is due to spatial location or the successional age of the study plots. Latitude and longitude, plot elevation (m above sea level), slope (°), and degree of ‘northness’ (cosine-transformed radian values of aspect) were assessed during plot establishment in 2008. Mean annual temperature and mean January and July temperatures per plot were obtained from continuous measurements with HOBO data loggers (one data logger in the center of each plot; 30 minutes time intervals from July 2011 to June 2012). Soil pH (measured potentiometrically in a H_2_O suspension), soil carbon (C) and nitrogen (N) content (measured with Vario ELIII elemental analyzer, Elementar, Hanau, Germany), and soil C:N ratio were determined from a bulk sample of nine soil cores (0–10 cm) per plot (taken in summer 2009^57^).

### Statistical analyses

All microorganism groups were assigned to the belowground compartment of the forest plots, together with the macrofaunal decomposers (diplopods and isopods, which are closely linked to the forest soil compartment, and which were analyzed as a single decomposer group due to low species numbers). All remaining arthropods were assigned to the aboveground compartment. Tree- and herb-layer plants were considered as the links between both compartments, as they can strongly determine both belowground (via roots, root deposition, woody debris, leaf litter) and aboveground (where all photosynthetically active parts of the plants are located) ecosystem structure. As specified below, we first tested for congruence in community patterns among the studied organism groups, and then constructed overall models of community relationships based on these congruence patterns.

#### Congruence in community patterns

Community congruence across organism groups was tested with Procrustes correlation analysis^24^ in the R package *vegan* ^64^, based on the site scores of principal components analysis (PCA) of species’ relative abundances. Standardizing the abundance data is necessary as sampling methods differ among organism groups and using raw species abundance data in PCA is not generally recommended^65^. Procrustes analysis has the advantage over simple linear correlation that it takes into account the full multivariate information of community data. It uses uniform scaling and rotation of the ordination solutions to minimize the sum of the squared residuals between data points of two datasets^24, 66^. Significance of the correlations was assessed with the *protest* permutation procedure^24, 64^, with 999 permutations. Statistical tests based on *protest* have been shown to be more powerful than Mantel tests for detecting community associations^24^. The expected number of false discoveries owing to multiple testing was calculated to adjust P-values^67^.

#### Modeling community relationships

We used structural equation models (SEM) in the R-package *lavaan* ^68^ to determine potentially causal links between the community patterns of the 21 organism groups we retained for analysis. Due to the large number of groups, and because relationships between the below- and aboveground organisms of our study can be assumed to be primarily mediated via the plant level, we conducted separate analyses for the below- and aboveground compartments, with tree- and herb layer plants as key structuring organisms included in all analyses. SEMs on community patterns were based on the site scores of the first two PCA axes for each organism group. These are the axes with the highest information content, representing the covariance structure of the species’ relative abundance patterns, and explaining on average 53.2% ± 16.5% SD of the total variance for the organism groups considered in our study. As similarities in community patterns among groups might be influenced by environmental conditions (either because of spatial variation or changes in these conditions during forest succession), we included the site scores of the first two axes of a principal components analysis on the 13 environmental and spatial parameters assessed for our study plots as covariables in the SEMs (Supplementary Table S2). Factoring out direct environmental effects allows interpreting the observed links between organism groups as effects of biotic interactions.

Because of the large number of potential links, we assembled the starting configuration of the initial SEM models in three steps: (i) we only considered relationships between organism groups with significant (P < 0.05) Procrustes correlations. Of these relationships, we (ii) only selected the most significant one out of the four possible correlation combinations between the first two PCA axes of the two groups. Finally, we used (iii) modification indices provided in the SEM output to identify missing pathways, that are required to obtain adequate model fit, and we added these pathways sequentially to the model until a non-significant global solution was achieved^25^. The same procedure was applied to selecting significant organism-environment links. Relationships across trophic levels (e.g. plants- >herbivores) were fit as direct causal pathways, relationships within trophic levels (e.g. between predators) as covariances.

The initial models were simplified through stepwise removal of uninformative paths, until a minimum adequate model was obtained, based on the resulting reduction in the Akaike Information Criterion (AIC). This minimum adequate model can be viewed as the most parsimonious solution to explaining community patterns across organism groups and trophic levels, where only the most influential relationships are retained^12^. Final model fits were indicated by a non-significant chi-square test (P > 0.05), low AIC, and low (<0.1) root mean square error of approximation (RMSEA, where values close to 0 indicate adequate model fit)^25^.

To determine whether the community webs were primarily bottom-up (i.e. by plants as resource suppliers and modifiers of both above- and belowground food webs^51^) or top-down (i.e. by consumers) controlled, we fit two alternative models for both the above- and belowground compartments. In the bottom-up model, the direction of the paths and thus the flow of causality was from the producer level (plants) to the higher trophic levels (bottom-up control). In the top-down model, path direction was reversed (top-down control, i.e. parasitoids->predators->herbivores->plants in the aboveground compartment). We considered fitting separate models most adequate to evaluate the support for overall bottom-up vs. top-down control. The relative support for bottom-up or top-down control was inferred by comparing the final models’ AIC values.

#### Diversity patterns

To compare relationships based on community patterns with those based on more frequently considered diversity metrics, we followed the same SEM procedure as described above (with the exception that we started the initial model with all relationships among organism groups that belonged to different trophic levels, i.e. with a less conservative approach than in the community analyses), with either species richness or Shannon diversity of the organism groups instead of community-data PCA site scores as the data source.

### Data availability

Data are available at http://china.befdata.biow.uni-leipzig.de/datasets/587.

## Electronic supplementary material

Supplementary PDF File

## References

[1] Ings TC (2009). Review: Ecological networks–beyond food webs. J. Anim. Ecol..

[2] Loreau, M. *From Populations to Ecosystems: Theoretical Foundations for a New Ecological Synthesis* (Princeton University Press, 2010).

[3] Naeem S, Duffy JE, Zavaleta E (2012). The functions of biological diversity in an age of extinction. Science.

[4] Thebault E, Huber V, Loreau M (2007). Cascading extinctions and ecosystem functioning: contrasting effects of diversity depending on food web structure. Oikos.

[5] Novotny V (2010). Guild-specific patterns of species richness and host specialization in plant-herbivore food webs from a tropical forest. J. Anim. Ecol..

[6] Tylianakis JM, Tscharntke T, Lewis OT (2007). Habitat modification alters the structure of tropical host-parasitoid food webs. Nature.

[7] Dunne JA (2013). Parasites affect food web structure primarily through increased diversity and complexity. PLoS Biol..

[8] Dunne JA, Williams RJ, Martinez ND (2002). Network structure and biodiversity loss in food webs: robustness increases with connectance. Ecol. Lett..

[9] Rzanny M, Kuu A, Voigt W (2013). Bottom-up and top-down forces structuring consumer communities in an experimental grassland. Oikos.

[10] Valverde A, Makhalanyane TP, Seely M, Cowan DA (2015). Cyanobacteria drive community composition and functionality in rock–soil interface communities. Mol. Ecol..

[11] Barberán A (2015). Relating belowground microbial composition to the taxonomic, phylogenetic, and functional trait distributions of trees in a tropical forest. Ecol. Lett..

[12] Scherber C (2010). Bottom-up effects of plant diversity on multitrophic interactions in a biodiversity experiment. Nature.

[13] Koricheva J, Mulder CPH, Schmid B, Joshi J, Huss-Danell K (2000). Numerical responses of different trophic groups of invertebrates to manipulations of plant diversity in grasslands. Oecologia.

[14] Srivastava DS, Vellend M (2005). Biodiversity-ecosystem function research: Is it relevant to conservation?. Annu. Rev. Ecol. Evol Syst..

[15] Stork, N. E. *et al*. Consistency of effects of tropical forest disturbance on species composition and richness relative to use of indicator taxa. *Conserv*. *Biol*., doi:10.1111/cobi.12883 (2017). 10.1111/cobi.12883 27982481

[16] Schaffers AP, Raemakers IP, Sýkora KV, Ter Braak CJ (2008). Arthropod assemblages are best predicted by plant species composition. Ecology.

[17] Perner J (2005). Effects of plant diversity, plant productivity and habitat parameters on arthropod abundance in montane European grasslands. Ecography.

[18] Barlow J (2007). Quantifying the biodiversity value of tropical primary, secondary, and plantation forests. Proc. Natl. Acad. Sci USA.

[19] Podgaiski LR (2013). Spider trait assembly patterns and resilience under fire-induced vegetation change in South Brazilian grasslands. PLoS ONE.

[20] Wu YT (2013). Forest age and plant species composition determine the soil fungal community composition in a Chinese subtropical forest. PLoS ONE.

[21] Schuldt A, Bruelheide H, Härdtle W, Assmann T (2012). Predator assemblage structure and temporal variability of species richness and abundance in forests of high tree diversity. Biotropica.

[22] Schmitz OJ (2008). Herbivory from individuals to ecosystems. Annu. Rev. Ecol. Evol Syst..

[23] Kéfi S (2012). More than a meal… integrating non-feeding interactions into food webs. Ecol. Lett..

[24] Peres-Neto PR, Jackson DA (2001). How well do multivariate data sets match? The advantages of a Procrustean superimposition approach over the Mantel test. Oecologia.

[25] Grace, J. B. *Structural Equation Modeling and Natural Systems* (Cambridge University Press, 2006).

[26] Schemske DW, Mittelbach GG, Cornell HV, Sobel JM, Roy K (2009). Is there a latitudinal gradient in the importance of biotic interactions?. Annu. Rev. Ecol. Evol Syst..

[27] Bagchi R (2014). Pathogens and insect herbivores drive rainforest plant diversity and composition. Nature.

[28] Liang M (2015). Arbuscular mycorrhizal fungi counteract the Janzen-Connell effect of soil pathogens. Ecology.

[29] Mangan SA (2010). Negative plant-soil feedback predicts tree-species relative abundance in a tropical forest. Nature.

[30] Bennett JA (2017). Plant-soil feedbacks and mycorrhizal type influence temperate forest population dynamics. Science.

[31] Husband R, Herre EA, Turner SL, Gallery R, Young JPW (2002). Molecular diversity of arbuscular mycorrhizal fungi and patterns of host association over time and space in a tropical forest. Mol. Ecol..

[32] Zhu Y, Comita LS, Hubbell SP, Ma K (2015). Conspecific and phylogenetic density‐dependent survival differs across life stages in a tropical forest. J. Ecol..

[33] Mitchell RJ (2010). Is vegetation composition or soil chemistry the best predictor of the soil microbial community?. Plant Soil.

[34] Schuldt A (2014). Woody plant phylogenetic diversity mediates bottom-up control of arthropod biomass in species-rich forests. Oecologia.

[35] Staab M (2016). Tree phylogenetic diversity promotes host–parasitoid interactions. Proc. R. Soc. B.

[36] Bezemer T (2006). Plant species and functional group effects on abiotic and microbial soil properties and plant–soil feedback responses in two grasslands. J. Ecol.

[37] Pini F, Galardini M, Bazzicalupo M, Mengoni A (2011). Plant-bacteria association and symbiosis: are there common genomic traits in Alphaproteobacteria?. Genes.

[38] Fierer N, Bradford MA, Jackson RB (2007). Toward and ecological classification of soil bacteria. Ecology.

[39] Schuldt A (2015). Multitrophic diversity in a biodiverse forest is highly nonlinear across spatial scales. Nat. Commun..

[40] Tedersoo L (2014). Global diversity and geography of soil fungi. Science.

[41] Both S (2011). Lack of tree layer control on herb layer characteristics in a subtropical forest, China. J. Veg. Sci..

[42] Simard SW (1997). Net transfer of carbon between ectomycorrhizal tree species in the field. Nature.

[43] van der Heijden MGA, Martin FM, Selosse M-A, Sanders IR (2015). Mycorrhizal ecology and evolution: the past, the present, and the future. New Phytol..

[44] Buscot F (2015). Implication of evolution and diversity in arbuscular and ectomycorrhizal symbioses. J. Plant Physiol..

[45] Mupepele A-C, Müller T, Dittrich M, Floren A (2014). Are temperate canopy spiders tree-species specific?. PLoS ONE.

[46] Staab M, Methorst J, Peters J, Blüthgen N, Klein A-M (2017). Tree diversity and nectar composition affect arthropod visitors on extrafloral nectaries in a diversity experiment. J. Plant Ecol..

[47] Schuldt A, Staab M (2015). Tree species richness strengthens relationships between ants and the functional composition of spider assemblages in a highly diverse forest. Biotropica.

[48] A’Bear AD, Johnson SN, Jones TH (2014). Putting the ‘upstairs–downstairs’ into ecosystem service: What can aboveground–belowground ecology tell us?. Biol. Control.

[49] Bardgett, R. D. & Wardle, D. A. *Aboveground-Belowground Linkages: Biotic Interactions*, *Ecosystem Processes*, *and Global Change* (Oxford University Press, 2010).

[50] Bezemer TM, van Dam NM (2005). Linking aboveground and belowground interactions via induced plant defenses. Trends Ecol. Evol..

[51] Wardle DA (2004). Ecological linkages between aboveground and belowground biota. Science.

[52] Ampoorter E (2015). Disentangling tree species identity and richness effects on the herb layer: first results from a German tree diversity experiment. J. Veg. Sci..

[53] Schuldt A (2011). Predator diversity and abundance provide little support for the enemies hypothesis in forests of high tree diversity. PLoS ONE.

[54] Staab M, Schuldt A, Assmann T, Klein AM (2014). Tree diversity promotes predator but not omnivore ants in a subtropical Chinese forest. Ecol. Entomol..

[55] Prober SM (2015). Plant diversity predicts beta but not alpha diversity of soil microbes across grasslands worldwide. Ecol. Lett..

[56] Hu Z, Yu M (2008). Study on successions sequence of evergreen broad-leaved forest in Gutian Mountain of Zhejiang, Eastern China: species diversity. Front. Biol. China.

[57] Bruelheide H (2011). Community assembly during secondary forest succession in a Chinese subtropical forest. Ecol. Monogr..

[58] Nacke H (2011). Pyrosequencing-based assessment of bacterial community structure along different management types in German forest and grassland soils. PLoS ONE.

[59] Wubet T (2012). Differences in soil fungal communities between European beech (*Fagus sylvatica* L.) dominated forests are related to soil and understory vegetation. PLoS ONE.

[60] Schloss PD (2009). Introducing mothur: open-source, platform-independent, community-supported software for describing and comparing microbial communities. Appl. Environ. Microbiol..

[61] Kunin V, Engelbrektson A, Ochman H, Hugenholtz P (2010). Wrinkles in the rare biosphere: pyrosequencing errors can lead to artificial inflation of diversity estimates. Environ. Microbiol.

[62] Huse SM (2008). Exploring microbial diversity and taxonomy using SSU rRNA hypervariable tag sequencing. PLoS Genet..

[63] Staab M, Ohl M, Zhu C-D, Klein A-M (2014). A unique nest-protection strategy in a new species of spider wasp. PLoS ONE.

[64] Oksanen, E. *et al*. Vegan: Community Ecology Package. R package version 2.0-9. Available from http://cran.r-project.org (2013).

[65] Borcard, D., Gillet, F. & Legendre, P. *Numerical ecology with R*. (Springer, 2011).

[66] Gower, J. *Statistical Methods of Comparing Different Multivariate Analyses of the Same Data* 138–149 (Edinburgh University Press, 1971).

[67] Benjamini Y, Hochberg Y (1995). Controlling the false discovery rate: a practical and powerful approach to multiple testing. J. R. Stat. Soc. B.

[68] Rosseel Y (2012). lavaan: and R package for structural equation modeling. J. Stat. Softw..

