# Supplementary material for: Belowground top-down and aboveground bottom-up effects structure multitrophic community relationships in a biodiverse forest
Source: Sci Rep. 2017 Jun 26;7:4222. doi: 10.1038/s41598-017-04619-3 (PMC5484685; doi:10.1038/s41598-017-04619-3)
Supplement: Supplementary file 1 — Supplementary PDF File [file 41598_2017_4619_MOESM1_ESM.pdf]

SUPPLEMENTARY MATERIAL

Belowground top-down and aboveground bottom-up effects structure multitrophic community relationships in a biodiverse forest

Andreas Schuldt, Helge Bruelheide, François Buscot, Thorsten Assmann, Alexandra Erfmeier, Alexandra-Maria Klein, Keping Ma, Thomas Scholten, Michael Staab, Christian Wirth, Jiayong Zhang, Tesfaye Wubet

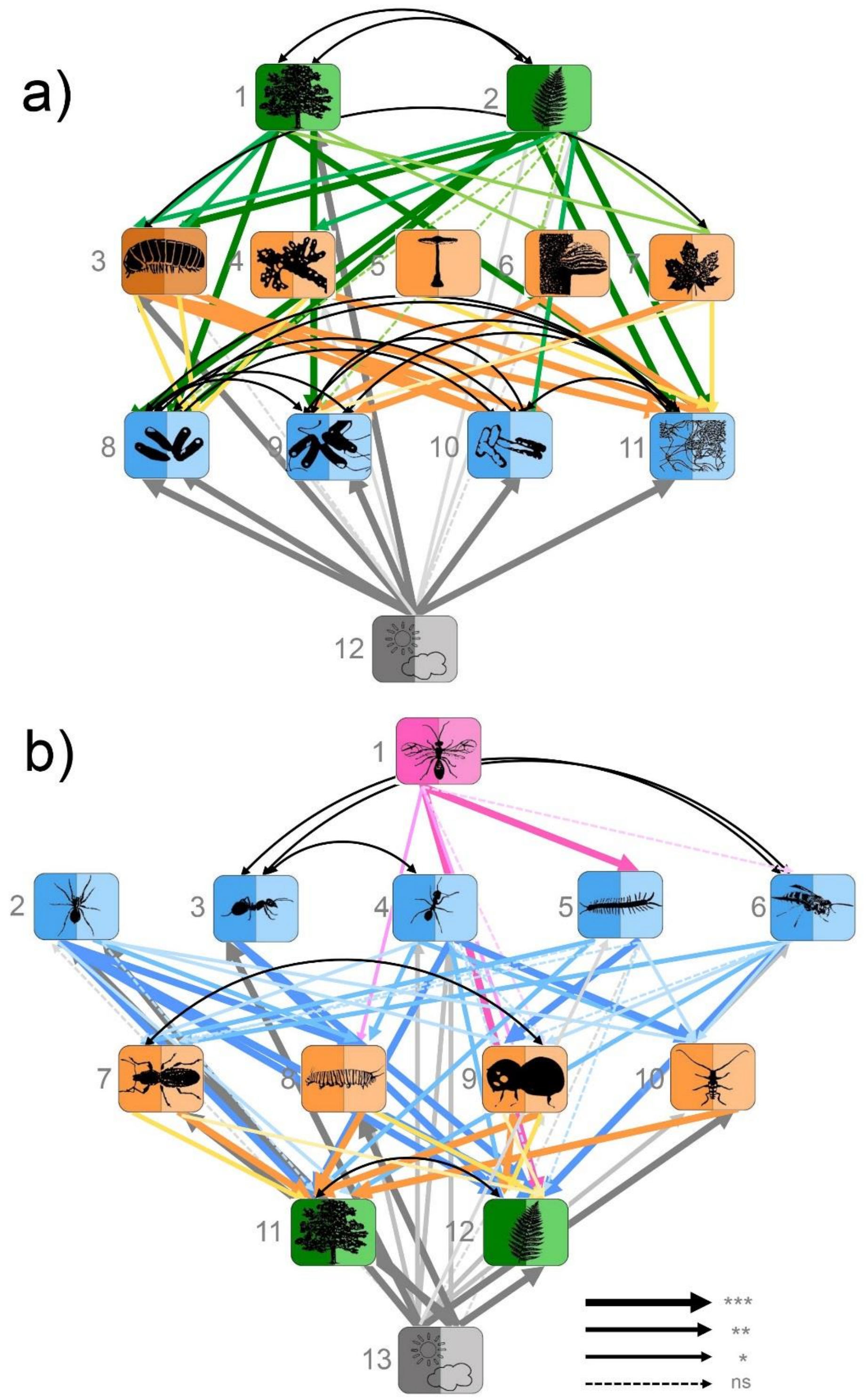

Figure S1 Community webs. Webs for a) *belowground* bottom-up control (χ^2 = 128.1, P = 0.054, DF = 104, RMSEA = 0.093, RMSEA P-value = 0.134, CFI = 0.943, AIC = -1452.7, N = 27) and b) *aboveground* top-down control (χ^2 = 191.4, P = 0.057, DF = 162, RMSEA = 0.082, RMSEA P-value = 0.167, CFI = 0.933, AIC = -324.6, N = 27). Structural equation models across trophic levels based on community structure, represented for each organism group by the first two axes of principal components analyses (PC1: darker shade, PC2: lighter shade) on species identities and relative abundances. Relationships are controlled for environmental dependencies, scaled proportional to their significance (*** P ≤ 0.001; ** P ≤

0.01; * P ≤ 0.05; ns nonsignificant). For clarity, only covariances ≤ 0.01 are plotted. See Tables S3 & S4 for detailed model ouput with path coefficients and error terms, and Figs S4 & S5 for an alternative presentation. Colors of boxes and corresponding arrows indicate different trophic or functional groups. Groups in a) are tree layer plants (1), herb layer plants (2), macrofaunal decomposers (3), arbuscular mycorrhizae (4), ectomycorrhizae (5), saprophytic fungi (6), pathogenic fungi (7), Acidobacteria (8), Alphaproteobacteria (9), Bacteriodetes (10), Chloroflexi (11), environment (12). Groups in b)are: Parasitic Hymenoptera (1), spiders (2), omnivorous ants (3), predatory ants (4), centipedes (5), predatory wasps (6), weevils (7), lepidopteran caterpillars (8), bark beetles (9), longhorn beetles (10), tree layer plants (11), herb layer plants (12), environment (13).

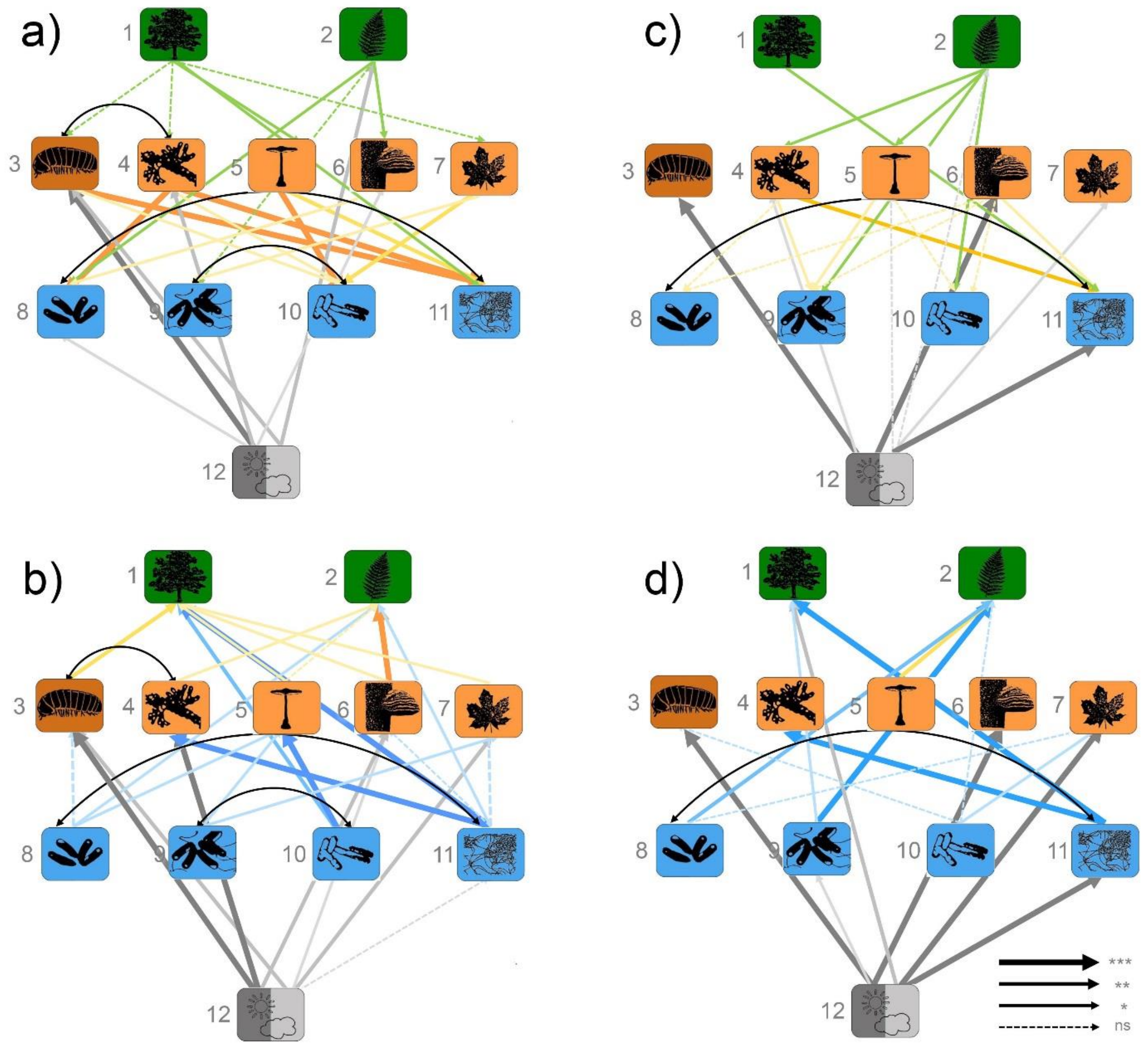

Figure S2 Belowground diversity webs. *Species richness* (a, b) and *Shannon diversity* (c, d) relationships for the *belowground* compartment. Upper row shows bottom-up control (a, c), lower row top-down control (b, d). Structural equation models across trophic levels based on species richness/Shannon diversity patterns (**a**: $\chi^2 = 48.1$, P = 0.470, DF = 48, RMSEA = 0.007, RMSEA P-value = 0.596, CFI = 1.0, AIC = 930.2; **b**: $\chi^2 = 47.5$, P = 0.454, DF = 47, RMSEA = 0.019, RMSEA P-value = 0.579, CFI = 0.997, AIC = 931.6; **c**: $\chi^2 = 56.1$, P = 0.886, DF = 70, RMSEA = 0.00, RMSEA P-value = 0.941, CFI = 1.0, AIC = 1063.4; **d**: $\chi^2 =$ 55.8, P = 0.855, DF = 68, RMSEA = 0.00, RMSEA P-value = 0.921, CFI = 1.0, AIC = 1067.1; N = 27 in all cases). Relationships are controlled for environmental dependencies, scaled proportional to their significance (*** $P \leq 0.001$; ** $P \leq 0.01$; * $P \leq 0.05$; ns nonsignificant). For clarity, only covariances ≤ 0.01 are plotted. See Tables S5 & S7 for detailed model ouput with path coefficients and error terms. Colors of boxes and corresponding arrows indicate different trophic or functional groups. Groups are: tree layer plants (1), herb layer plants (2), macrofaunal decomposers (3), arbuscular mycorrhizae (4), ectomycorrhizae (5), saprophytic fungi (6), pathogenic fungi (7), Acidobacteria (8), Alphaproteobacteria (9), Bacteriodetes (10), Chloroflexi (11), environment (12).

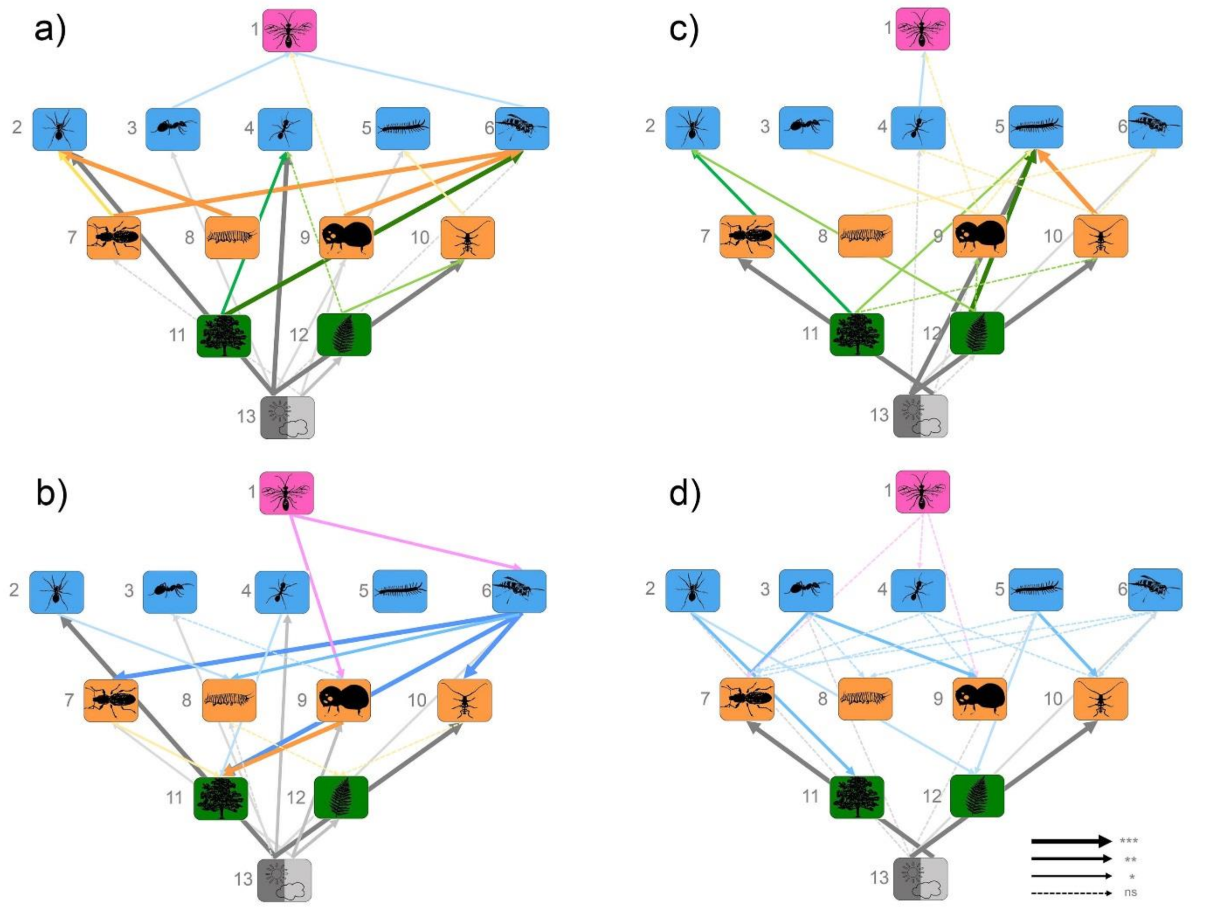

Figure S3 Aboveground diversity webs. *Species richness* (a, b) and *Shannon diversity* (c, d) relationships for the *aboveground* compartment. Upper row shows bottom-up control (a, c), lower row top-down control (b, d). Structural equation models across trophic levels based on species richness/Shannon diversity patterns (**a**: χ^2 = 63.4, P = 0.666, DF = 69, RMSEA = 0.00, RMSEA P-value = 0.788, CFI = 1.0, AIC = 1029.6; **b**: χ^2 = 69.2, P = 0.470, DF = 69, RMSEA = 0.011, RMSEA P-value = 0.620, CFI = 0.998, AIC = 1035.4; **c**: χ^2 = 56.1, P = 0.886, DF = 70, RMSEA = 0.00, RMSEA P-value = 0.941, CFI = 1.0, AIC = 1063.4; **d**: χ^2 = 55.8, P = 0.855, DF = 68, RMSEA = 0.00, RMSEA P-value = 0.921, CFI = 1.0, AIC = 1067.1; N = 27 in all cases). Relationships are controlled for environmental dependencies, scaled proportional to their significance (*** $P \leq 0.001$; ** $P \leq 0.01$; * $P \leq 0.05$; ns nonsignificant). For clarity, only covariances ≤ 0.01 are plotted. See Tables S6 & S8 for detailed model ouput with path coefficients and error terms. Colors of boxes and corresponding arrows indicate different trophic or functional groups. Groups are: Parasitic Hymenoptera (1), spiders (2), omnivorous ants (3), predatory ants (4), centipedes (5), predatory wasps (6), weevils (7), lepidopteran caterpillars (8), bark beetles (9), longhorn beetles (10), tree layer plants (11), herb layer plants (12), environment (13).

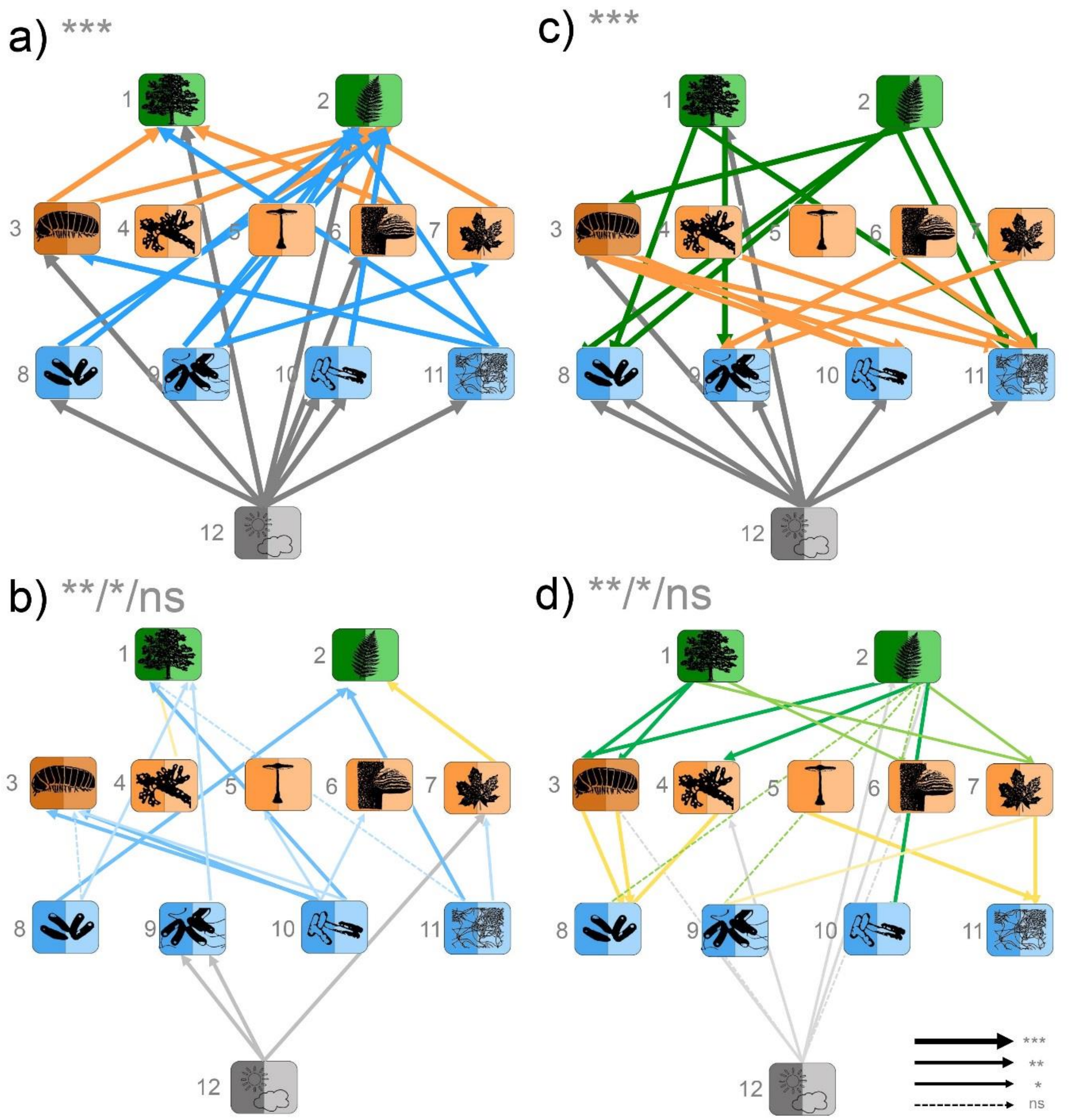

Figure S4 Belowground community webs separated by significance. Panels a and b correspond to Figure 1 (top-down control), panles c and d correspond to Figure S1a (bottom-up control). For a better overview, the overall relationships were plotted separately for relationships with $P < 0.001$ (a and c) and $P > 0.001$ (b and d). For further details, see Figs. 1 and S1. Colors of boxes and corresponding arrows indicate different trophic or functional groups. Groups are: tree layer plants (1), herb layer plants (2), macrofaunal decomposers (3), arbuscular mycorrhizae (4), ectomycorrhizae (5), saprophytic fungi (6), pathogenic fungi (7), Acidobacteria (8), Alphaproteobacteria (9), Bacteriodetes (10), Chloroflexi (11), environment (12).

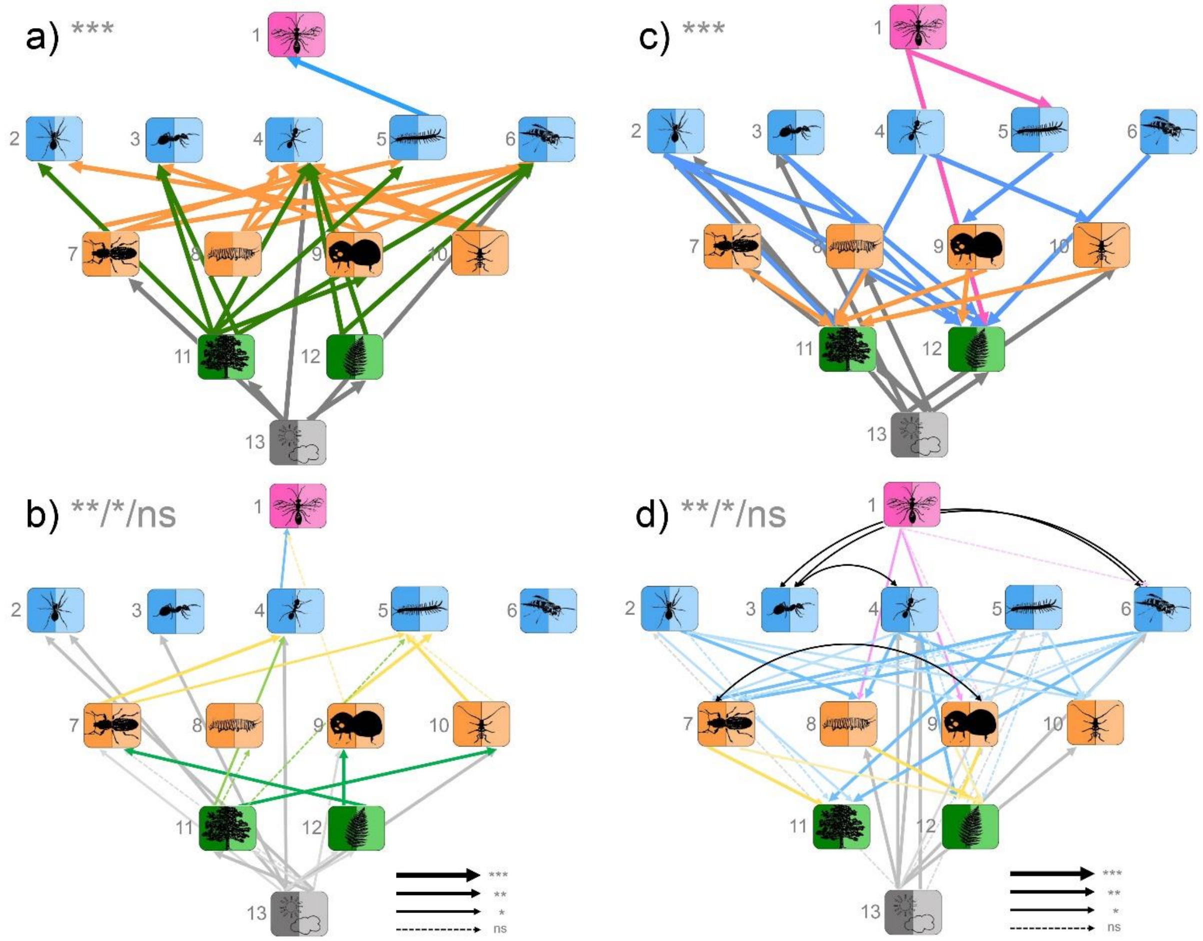

Figure S5 Aboveground community webs separated by significance. Panels a and b correspond to Figure 2 (bottom-up control), panles c and d correspond to Figure S1b (top-down control). For a better overview, the overall relationships were plotted separately for relationships with P < 0.001 (a and c) and P > 0.001 (b and d). For further details, see Figs. 1 and S1. Colors of boxes and corresponding arrows indicate different trophic or functional groups. Groups are: Parasitic Hymenoptera (1), spiders (2), omnivorous ants (3), predatory ants (4), centipedes (5), predatory wasps (6), weevils (7), lepidopteran caterpillars (8), bark beetles (9), longhorn beetles (10), tree layer plants (11), herb layer plants (12), environment (13).

Table S1. Congruence of community patterns across trophic levels. Procrustes correlation results (top-right: correlation coefficients, bottom-left: significance values[1] of community similarity among all above- and belowground organism groups based on principal components analysis (PCA) of species identities and relative abundances. Bacterial groups not considered in the structural equation models are in grey.

	Acidobecteria	Alphaproteobact.	Actinomycetes	Gammaproteobac.	Deltaproteobac.	Chloroflexi	Betaproteobac.	Bacteroidetes	Parasitoids	Spiders	Ants (pred.)	Ants (omni.)	Centipedes	Wasps (pred.)	Weevils	Bark beetles	Lepidoptera	Longhorn beetles	Tree layer plants	Herb layer plants	Decomposers	AMF	ECM	Saprophytic fungi	Pathogenic fungi
Acidobecteria		0.80	0.79	0.78	0.67	0.87	0.64	0.80	0.56	0.64	0.56	0.65	0.67	0.45	0.55	0.59	0.69	0.61	0.72	0.61	0.63	0.69	0.73	0.77	0.69
Alphaproteobact.	**0.001**		0.76	0.72	0.64	0.81	0.64	0.77	0.62	0.57	0.48	0.56	0.64	0.48	0.49	0.55	0.64	0.60	0.65	0.59	0.63	0.67	0.68	0.72	0.70
Actinomycetes	**0.001**	**0.001**		0.74	0.66	0.84	0.65	0.77	0.52	0.62	0.51	0.65	0.67	0.47	0.53	0.54	0.66	0.65	0.73	0.63	0.66	0.70	0.69	0.73	0.68
Gammaproteobac.	**0.001**	**0.001**	**0.001**		0.60	0.76	0.68	0.78	0.46	0.51	0.44	0.54	0.54	0.51	0.45	0.55	0.59	0.58	0.63	0.59	0.65	0.61	0.63	0.67	0.61
Deltaproteobac.	**0.001**	**0.004**	**0.002**	**0.009**		0.67	0.61	0.68	0.46	0.54	0.51	0.56	0.60	0.40	0.51	0.49	0.65	0.54	0.61	0.50	0.52	0.67	0.68	0.68	0.64
Chloroflexi	**0.001**	**0.001**	**0.001**	**0.001**	**0.010**		0.63	0.80	0.59	0.68	0.56	0.71	0.71	0.48	0.60	0.63	0.77	0.67	0.77	0.70	0.69	0.76	0.79	0.84	0.76
Betaproteobac.	**0.001**	**0.001**	**0.001**	**0.001**	**0.004**	**0.003**		0.71	0.44	0.50	0.45	0.49	0.54	0.46	0.37	0.50	0.58	0.56	0.57	0.50	0.54	0.56	0.61	0.62	0.56
Bacteroidetes	**0.001**	**0.001**	**0.001**	**0.001**	**0.002**	**0.001**	**0.001**		0.51	0.60	0.53	0.59	0.65	0.46	0.52	0.59	0.68	0.63	0.70	0.60	0.67	0.68	0.74	0.76	0.72
Parasitoids	0.184	**0.001**	0.512	0.629	0.883	0.186	0.800	0.841		0.51	0.46	0.50	0.64	0.47	0.51	0.51	0.61	0.50	0.59	0.45	0.47	0.63	0.63	0.64	0.67
Spiders	**0.001**	**0.034**	**0.005**	0.110	0.126	**0.001**	0.116	**0.016**	0.283		0.58	0.66	0.61	0.46	0.50	0.53	0.63	0.53	0.74	0.57	0.54	0.63	0.63	0.65	0.63
Ants (pred.)	**0.005**	0.115	**0.039**	0.209	**0.018**	**0.003**	0.184	**0.017**	0.274	**0.003**		0.58	0.54	0.36	0.43	0.43	0.53	0.52	0.53	0.42	0.47	0.48	0.51	0.53	0.47
Ants (omni.)	**0.003**	0.132	**0.001**	0.067	0.060	**0.001**	0.291	0.112	0.648	**0.001**	**0.001**		0.64	0.50	0.52	0.60	0.66	0.63	0.73	0.66	0.49	0.63	0.65	0.69	0.63
Centipedes	**0.031**	0.052	**0.011**	0.559	0.284	0.057	0.251	0.137	**0.006**	**0.015**	**0.012**	**0.007**		0.47	0.61	0.59	0.75	0.67	0.71	0.58	0.53	0.74	0.77	0.76	0.74
Wasps (pred.)	0.186	0.052	0.055	**0.010**	0.476	0.063	0.069	0.154	0.087	0.097	0.611	**0.016**	0.187		0.36	0.46	0.44	0.46	0.52	0.45	0.44	0.48	0.46	0.48	0.43
Weevils	0.092	0.489	0.196	0.524	0.193	0.051	0.985	0.368	0.141	0.165	0.324	0.136	**0.005**	0.655		0.45	0.59	0.50	0.56	0.53	0.55	0.59	0.60	0.62	0.58
Bark beetles	**0.016**	0.094	0.204	**0.031**	0.550	**0.010**	0.163	**0.042**	0.402	0.099	0.461	**0.005**	0.095	0.126	0.660		0.60	0.54	0.61	0.55	0.51	0.60	0.64	0.64	0.64
Lepidoptera	0.419	0.839	0.561	0.754	0.396	0.306	0.346	0.912	0.525	0.103	**0.048**	0.056	0.217	0.654	0.350	0.501		0.70	0.74	0.61	0.56	0.83	0.87	0.87	0.78
Longhorn beetles	0.127	0.105	**0.007**	**0.043**	0.582	**0.036**	**0.050**	**0.048**	0.825	0.367	**0.031**	**0.010**	**0.031**	0.177	0.467	0.295	0.181		0.67	0.55	0.55	0.71	0.72	0.72	0.70
Tree layer plants	**0.001**	**0.014**	**0.001**	**0.004**	0.103	**0.001**	0.061	**0.003**	0.138	**0.001**	**0.015**	**0.001**	**0.003**	**0.009**	0.115	**0.019**	0.096	**0.025**		0.70	0.58	0.74	0.76	0.80	0.74
Herb layer plants	**0.017**	**0.019**	**0.001**	**0.007**	0.444	**0.001**	0.135	**0.019**	0.866	**0.019**	0.400	**0.001**	0.149	0.099	0.051	**0.047**	0.332	0.203	**0.001**		0.56	0.62	0.66	0.65	0.61
Decomposers	**0.003**	**0.001**	**0.001**	**0.001**	0.097	**0.001**	**0.015**	**0.001**	0.440	**0.034**	0.085	0.265	0.283	0.082	**0.017**	0.097	0.460	0.080	**0.025**	**0.006**		0.58	0.58	0.63	0.57
AMF	0.362	0.232	**0.026**	0.246	0.066	0.248	0.677	0.733	0.152	0.089	0.681	0.513	0.364	0.216	0.313	0.489	0.334	0.061	**0.029**	0.216	0.215		0.85	0.83	0.81
ECM	0.066	0.539	0.552	0.122	0.230	0.455	0.105	**0.033**	0.440	0.165	0.225	0.573	0.341	0.489	0.619	0.168	0.237	0.135	0.084	**0.006**	0.306	0.652		0.90	0.85
Saprophytic fungi	**0.001**	**0.001**	**0.001**	**0.001**	0.073	**0.001**	**0.013**	**0.002**	0.110	**0.009**	**0.040**	**0.005**	0.186	0.245	0.105	0.055	0.092	0.075	**0.001**	**0.031**	**0.004**	0.864	0.275		0.84
Pathogenic fungi	0.170	**0.018**	0.096	0.096	0.306	0.109	0.490	**0.009**	**0.002**	0.053	0.670	0.230	0.154	0.701	0.403	**0.012**	0.955	0.106	0.068	0.236	0.196	0.365	0.179	0.282	

[1]original P-values returned from the Procrustes correlation analyses are reported here. When adapting P-values to the expected number of false discoveries owing to multiple testing, P-values > 0.015 turn non-significant (> 0.05).

Table S2. Component loadings of environmental PCA. Loadings and eigenvalues of principal components (PC) selected from PCA reduction analysis on environmental variables (most influential variables in bold)

	PC1	PC2
Elevation	**0.41**	-0.12
Slope	0.04	**0.39**
Northness	0.15	0.14
Eastness	-0.12	0.04
Latitude	**0.27**	**-0.26**
Longitude	0.13	**0.32**
Soil pH	-0.22	-0.22
Soil N-content	**0.29**	**0.42**
Soil C-content	**0.27**	**0.49**
Soil C:N-ratio	-0.13	**0.26**
Mean January temperature	**-0.42**	0.14
Mean July temperature	**-0.37**	0.24
Mean annual temperature	**-0.41**	0.20
Eigenvalue	2.22	1.56

Table S3. Structural equation model results for *belowground community structure.* a) *bottom-up* control and b) *top-down* control (see Figure 1 & S1). Abbreviations: acido=Acidobacteria, alpha=Alphaproteobacteria, amf=Arbuscular mycorrhizal fungi, bacte=Bacteroidetes, chloroflexi=Chloroflexi, decomp=Macrofaunal decomposers, ecm=Ectomycorrhizal fungi,envir=Environment, herb=Herb layer plants, patho=Pathogenic fungi, sapro=Saprophytic fungi, tree=Tree layer plants

**a) bottom-up**									
**Left hand side**	**Operator**	**Right hand side**	**Estimate**	**SE**	**z**	**P**	**CI lower**	**CI upper**	**Standard. Est.**
Taxon relations									
bacte1	~	decomp1	0.152	0.039	3.907	0.000	0.076	0.228	0.513
bacte2	~	decomp2	-0.130	0.032	-4.080	0.000	-0.193	-0.068	-0.356
bacte1	~	decomp2	0.255	0.064	3.957	0.000	0.129	0.381	0.490
acido2	~	herb1	0.033	0.009	3.639	0.000	0.015	0.050	0.420
chloroflexi1	~	herb1	0.043	0.007	5.792	0.000	0.029	0.058	0.257
chloroflexi2	~	herb2	0.083	0.017	4.877	0.000	0.049	0.116	0.592
alpha2	~	patho2	-0.084	0.018	-4.791	0.000	-0.118	-0.050	-0.509
chloroflexi2	~	sapro1	0.281	0.064	4.412	0.000	0.156	0.406	0.485
alpha1	~	sapro1	-0.288	0.076	-3.789	0.000	-0.437	-0.139	-0.375
chloroflexi2	~	tree1	0.121	0.021	5.826	0.000	0.080	0.161	0.576
chloroflexi2	~	amf2	-0.075	0.023	-3.273	0.001	-0.120	-0.030	-0.333
chloroflexi1	~	decomp1	-0.043	0.013	-3.400	0.001	-0.068	-0.018	-0.193
acido1	~	herb1	0.026	0.008	3.333	0.001	0.011	0.042	0.223
decomp2	~	herb2	0.319	0.094	3.378	0.001	0.134	0.504	0.541
acido2	~	tree1	-0.062	0.019	-3.244	0.001	-0.099	-0.024	-0.384
alpha1	~	tree2	-0.126	0.039	-3.197	0.001	-0.203	-0.049	-0.331
chloroflexi2	~	ecm1	-0.072	0.024	-3.024	0.002	-0.118	-0.025	-0.233
bacte2	~	herb2	0.068	0.022	3.149	0.002	0.026	0.111	0.317
acido2	~	decomp2	-0.059	0.019	-3.017	0.003	-0.097	-0.021	-0.324
acido2	~	amf2	-0.044	0.016	-2.845	0.004	-0.075	-0.014	-0.256
decomp1	~	herb1	-0.302	0.105	-2.883	0.004	-0.508	-0.097	-0.402
decomp1	~	tree1	0.682	0.239	2.855	0.004	0.214	1.150	0.439
decomp2	~	tree1	0.353	0.124	2.859	0.004	0.111	0.596	0.399
acido2	~	decomp1	-0.039	0.014	-2.825	0.005	-0.066	-0.012	-0.376
amf2	~	herb2	0.289	0.108	2.670	0.008	0.077	0.501	0.465
chloroflexi2	~	patho2	0.053	0.020	2.649	0.008	0.014	0.093	0.272
patho2	~	herb2	-0.240	0.103	-2.325	0.020	-0.442	-0.038	-0.337
alpha1	~	patho2	0.058	0.027	2.149	0.032	0.005	0.111	0.224
patho2	~	tree1	0.374	0.180	2.078	0.038	0.021	0.727	0.350
sapro1	~	tree2	-0.184	0.091	-2.016	0.044	-0.364	-0.005	-0.372
alpha1	~	herb2	-0.036	0.020	-1.838	0.066	-0.075	0.002	-0.197
acido1	~	herb2	0.021	0.013	1.659	0.097	-0.004	0.046	0.129
sapro1	~	herb2	-0.052	0.034	-1.535	0.125	-0.118	0.014	-0.215
Environmental effects									
acido2	~	envir1	-0.495	0.132	-3.756	0.000	-0.753	-0.237	-0.444
acido1	~	envir1	0.931	0.261	3.561	0.000	0.419	1.443	0.554
alpha2	~	envir1	0.705	0.165	4.271	0.000	0.381	1.028	0.577

chloroflexi1	~	envir1	1.370	0.272	5.045	0.000	0.838	1.902	0.567
bacte2	~	envir1	-1.193	0.303	-3.939	0.000	-1.786	-0.599	-0.532
tree2	~	envir1	-3.627	0.678	-5.352	0.000	-4.955	-2.299	-0.717
decomp1	~	envir1	-5.429	1.302	-4.170	0.000	-7.980	-2.877	-0.504
herb2	~	envir1	-4.129	1.837	-2.248	0.025	-7.729	-0.528	-0.397
alpha1	~	envir1	0.688	0.326	2.109	0.035	0.049	1.327	0.358
amf2	~	envir1	2.312	1.125	2.056	0.040	0.108	4.516	0.358
herb1	~	envir1	4.253	2.126	2.001	0.045	0.087	8.420	0.297
sapro1	~	envir1	0.828	0.432	1.920	0.055	-0.017	1.674	0.331
decomp2	~	envir1	1.484	0.931	1.594	0.111	-0.341	3.309	0.242
Variances and covariances									
acido1	~~	acido1	0.001	0.000	4.492	0.000	0.000	0.001	0.617
acido2	~~	acido2	0.000	0.000	4.325	0.000	0.000	0.000	0.443
alpha1	~~	alpha1	0.001	0.000	4.199	0.000	0.001	0.002	0.635
alpha2	~~	alpha2	0.000	0.000	3.674	0.000	0.000	0.001	0.487
amf2	~~	amf2	0.016	0.004	3.729	0.000	0.007	0.024	0.788
bacte1	~~	bacte1	0.002	0.001	3.674	0.000	0.001	0.003	0.463
bacte2	~~	bacte2	0.001	0.000	3.674	0.000	0.000	0.002	0.446
alpha1	~~	chloroflexi1	0.001	0.000	3.634	0.000	0.000	0.001	0.734
chloroflexi1	~~	chloroflexi1	0.001	0.000	4.526	0.000	0.001	0.001	0.322
acido1	~~	chloroflexi1	0.001	0.000	4.169	0.000	0.000	0.001	0.841
chloroflexi2	~~	chloroflexi2	0.000	0.000	3.674	0.000	0.000	0.000	0.257
decomp1	~~	decomp1	0.026	0.007	3.674	0.000	0.012	0.040	0.470
decomp2	~~	decomp2	0.011	0.003	3.740	0.000	0.005	0.016	0.594
ecm1	~~	ecm1	0.011	0.003	3.674	0.000	0.005	0.016	1.000
envir.new1	~~	envir1	0.000	0.000	3.674	0.000	0.000	0.001	1.000
herb1	~~	herb1	0.089	0.022	4.067	0.000	0.046	0.132	0.912
herb2	~~	herb2	0.043	0.012	3.674	0.000	0.020	0.066	0.842
patho2	~~	patho2	0.020	0.005	3.816	0.000	0.010	0.030	0.764
sapro1	~~	sapro1	0.001	0.000	3.674	0.000	0.001	0.002	0.389
tree1	~~	tree1	0.023	0.006	3.674	0.000	0.011	0.035	1.000
tree2	~~	tree2	0.006	0.002	3.855	0.000	0.003	0.009	0.485
alpha1	~~	acido1	0.001	0.000	3.457	0.001	0.000	0.001	0.699
acido1	~~	alpha2	0.000	0.000	3.359	0.001	0.000	0.000	0.509
bacte2	~~	chloroflexi1	-0.001	0.000	-3.224	0.001	-0.001	0.000	-0.687
acido2	~~	bacte1	0.000	0.000	-3.084	0.002	-0.001	0.000	-0.590
acido2	~~	chloroflexi1	0.000	0.000	3.074	0.002	0.000	0.000	0.279
alpha2	~~	chloroflexi1	0.000	0.000	3.137	0.002	0.000	0.000	0.401
acido1	~~	bacte2	-0.001	0.000	-2.990	0.003	-0.001	0.000	-0.606
herb1	~~	tree1	0.027	0.009	2.944	0.003	0.009	0.044	0.590
tree2	~~	herb1	-0.011	0.004	-2.789	0.005	-0.019	-0.003	-0.479
patho2	~~	decomp1	0.012	0.005	2.604	0.009	0.003	0.022	0.542
alpha1	~~	bacte2	-0.001	0.000	-2.474	0.013	-0.001	0.000	-0.493
acido2	~~	alpha1	0.000	0.000	2.352	0.019	0.000	0.000	0.234
bacte1	~~	chloroflexi1	0.000	0.000	-2.198	0.028	0.000	0.000	-0.183
tree2	~~	herb2	0.006	0.003	2.123	0.034	0.000	0.011	0.360
acido2	~~	chloroflexi2	0.000	0.000	-2.068	0.039	0.000	0.000	-0.309

decomp2	~~	patho2	0.005	0.002	2.010	0.044	0.000	0.010	0.332
amf2	~~	ecm1	-0.005	0.002	-1.829	0.067	-0.009	0.000	-0.354
amf2	~~	decomp2	-0.004	0.002	-1.779	0.075	-0.008	0.000	-0.313
alpha1	~~	chloroflexi2	0.000	0.000	1.731	0.083	0.000	0.000	0.160

b) top-down

Left hand side	Operator	Right hand side	Estimate	SE	z	P	CI lower	CI upper	Standard. Est.
Taxon relations									
herb2	~	acido1	2.830	0.606	4.672	0.000	1.643	4.017	0.380
herb1	~	acido2	6.950	1.199	5.796	0.000	4.600	9.300	0.419
herb1	~	alpha1	-3.237	0.503	-6.431	0.000	-4.223	-2.250	-0.295
herb2	~	alpha1	-2.216	0.402	-5.510	0.000	-3.005	-1.428	-0.306
herb1	~	alpha2	-10.434	1.214	-8.593	0.000	-12.814	-8.054	-0.696
patho2	~	alpha2	-3.684	0.822	-4.482	0.000	-5.295	-2.073	-0.676
herb2	~	amf2	0.695	0.102	6.800	0.000	0.495	0.896	0.349
herb2	~	bacte2	2.055	0.458	4.485	0.000	1.157	2.953	0.377
decomp2	~	chloroflexi2	2.179	0.618	3.526	0.000	0.968	3.390	0.499
herb1	~	chloroflexi2	6.751	0.819	8.245	0.000	5.146	8.356	0.494
tree1	~	chloroflexi2	3.290	0.458	7.182	0.000	2.392	4.188	0.670
tree1	~	decomp1	0.209	0.054	3.882	0.000	0.103	0.314	0.351
herb2	~	decomp2	0.658	0.122	5.375	0.000	0.418	0.898	0.318
herb2	~	ecm1	0.797	0.137	5.802	0.000	0.527	1.066	0.304
tree2	~	sapro1	-0.935	0.226	-4.141	0.000	-1.378	-0.492	-0.435
herb2	~	sapro1	-1.855	0.333	-5.574	0.000	-2.508	-1.203	-0.382
herb1	~	patho2	-0.493	0.155	-3.176	0.001	-0.798	-0.189	-0.179
decomp2	~	bacte1	1.217	0.392	3.105	0.002	0.449	1.985	0.674
tree1	~	bacte2	-1.136	0.381	-2.982	0.003	-1.883	-0.389	-0.383
decomp1	~	bacte1	1.296	0.448	2.892	0.004	0.418	2.174	0.379
herb1	~	acido1	3.461	1.224	2.828	0.005	1.062	5.860	0.307
herb1	~	chloroflexi1	3.209	1.167	2.750	0.006	0.922	5.497	0.431
herb2	~	patho2	-0.258	0.100	-2.580	0.010	-0.454	-0.062	-0.142
tree1	~	amf2	-0.188	0.074	-2.533	0.011	-0.333	-0.042	-0.173
tree2	~	acido2	-1.202	0.481	-2.502	0.012	-2.144	-0.260	-0.248
patho2	~	chloroflexi2	1.575	0.643	2.451	0.014	0.316	2.835	0.317
tree2	~	alpha2	1.091	0.496	2.199	0.028	0.118	2.063	0.249
ecm1	~	bacte1	0.527	0.255	2.066	0.039	0.027	1.027	0.369
decomp2	~	bacte2	-0.798	0.391	-2.042	0.041	-1.564	-0.032	-0.303
sapro1	~	bacte1	-0.210	0.103	-2.030	0.042	-0.412	-0.007	-0.273
tree1	~	chloroflexi1	0.704	0.405	1.737	0.082	-0.090	1.498	0.263
decomp2	~	acido2	1.661	1.103	1.506	0.132	-0.501	3.823	0.314
Environmental effects									
acido1	~	envir1	0.992	0.257	3.867	0.000	0.489	1.495	0.597
chloroflexi1	~	envir1	1.985	0.270	7.361	0.000	1.457	2.514	0.788
bacte2	~	envir1	-1.530	0.323	-4.739	0.000	-2.163	-0.897	-0.674
tree2	~	envir1	-2.882	0.745	-3.867	0.000	-4.342	-1.421	-0.526
sapro1	~	envir1	1.564	0.330	4.746	0.000	0.918	2.211	0.613

bacte1	~	envir1	-1.259	0.369	-3.413	0.001	-1.982	-0.536	-0.379
herb1	~	envir1	6.646	1.947	3.413	0.001	2.830	10.462	0.354
decomp1	~	envir1	-5.442	1.627	-3.345	0.001	-8.631	-2.254	-0.480
alpha2	~	envir1	0.643	0.206	3.113	0.002	0.238	1.047	0.514
alpha1	~	envir1	0.793	0.291	2.724	0.006	0.223	1.364	0.464
patho2	~	envir1	2.978	1.112	2.679	0.007	0.799	5.156	0.437
Variances and covariances									
acido1	~~	acido1	0.001	0.000	3.958	0.000	0.000	0.001	0.644
acido2	~~	acido2	0.001	0.000	3.800	0.000	0.000	0.001	1.000
alpha1	~~	alpha1	0.001	0.000	3.674	0.000	0.001	0.002	0.784
alpha2	~~	alpha2	0.001	0.000	3.674	0.000	0.000	0.001	0.736
amf2	~~	amf2	0.018	0.005	3.674	0.000	0.009	0.028	1.000
bacte1	~~	bacte1	0.004	0.001	3.674	0.000	0.002	0.007	0.856
bacte2	~~	bacte2	0.001	0.000	3.674	0.000	0.001	0.002	0.546
chloroflexi1	~~	chloroflexi1	0.001	0.000	4.545	0.000	0.001	0.002	0.380
acido1	~~	chloroflexi1	0.001	0.000	3.744	0.000	0.000	0.001	0.788
chloroflexi2	~~	chloroflexi2	0.001	0.000	3.674	0.000	0.000	0.001	1.000
decomp1	~~	decomp1	0.030	0.008	3.674	0.000	0.014	0.046	0.488
decomp2	~~	decomp2	0.010	0.003	3.674	0.000	0.005	0.015	0.576
ecm1	~~	ecm1	0.009	0.002	3.674	0.000	0.004	0.014	0.864
envir.new1	~~	envir1	0.000	0.000	3.674	0.000	0.000	0.001	1.000
herb1	~~	herb1	0.021	0.005	4.118	0.000	0.011	0.030	0.123
herb2	~~	herb2	0.005	0.001	3.674	0.000	0.002	0.008	0.071
patho2	~~	patho2	0.012	0.003	3.674	0.000	0.006	0.019	0.556
sapro1	~~	sapro1	0.001	0.000	3.674	0.000	0.001	0.002	0.422
tree1	~~	tree1	0.005	0.001	3.674	0.000	0.002	0.008	0.235
tree2	~~	tree2	0.004	0.001	3.674	0.000	0.002	0.006	0.255
bacte1	~~	acido2	-0.001	0.000	-3.199	0.001	-0.002	0.000	-0.763
chloroflexi1	~~	acido2	0.000	0.000	3.136	0.002	0.000	0.001	0.421
chloroflexi1	~~	bacte2	-0.001	0.000	-3.141	0.002	-0.001	0.000	-0.632
tree2	~~	herb1	-0.005	0.002	-2.950	0.003	-0.008	-0.002	-0.572
tree1	~~	herb1	0.006	0.002	2.924	0.003	0.002	0.010	0.558
acido1	~~	bacte2	-0.001	0.000	-2.808	0.005	-0.001	0.000	-0.570
bacte1	~~	chloroflexi1	-0.001	0.000	-2.659	0.008	-0.001	0.000	-0.340
acido1	~~	alpha2	0.000	0.000	2.545	0.011	0.000	0.001	0.462
alpha2	~~	chloroflexi1	0.000	0.000	2.455	0.014	0.000	0.000	0.345
patho2	~~	decomp1	0.008	0.004	2.063	0.039	0.000	0.016	0.433
acido2	~~	chloroflexi2	0.000	0.000	-1.917	0.055	0.000	0.000	-0.215
decomp2	~~	sapro1	-0.001	0.001	-1.897	0.058	-0.003	0.000	-0.392

Table S4. Structural equation model results for *aboveground community structure.* a) *bottom-up* control and b) *top-down* control (see Figure 2 & S1). Abbreviations: ant_omni=Omnivorous ants, ant_pred=Predatory ants, ceramby=Longhorn beetles, chilo=Centipedes, envir=Environment, herb=Herb layer plants, lepi=Lepidoptera, para=Parasitoids, scoly=Bark beetles, spider=Spiders, tree=Tree layer plants, wasp=Wasps, weevil=Weevils

**a) bottom-up**									
**Left hand side**	**Operator**	**Right hand side**	**Estimate**	**SE**	**z**	**P**	**CI lower**	**CI upper**	**Standard. Est.**
Taxon relations									
ant_pred2	~	ceramby1	-0.306	0.082	-3.730	0.000	-0.466	-0.145	-0.193
spider2	~	ceramby2	0.289	0.077	3.738	0.000	0.137	0.440	0.469
ant_pred1	~	ceramby2	1.341	0.233	5.749	0.000	0.884	1.799	0.681
para1	~	chilo2	-1.390	0.342	-4.064	0.000	-2.061	-0.720	-0.696
wasp1	~	herb1	-0.321	0.090	-3.574	0.000	-0.497	-0.145	-0.286
ant_pred2	~	herb1	0.797	0.090	8.867	0.000	0.621	0.973	0.685
ant_pred2	~	herb2	-0.519	0.079	-6.559	0.000	-0.675	-0.364	-0.329
ant_pred2	~	lepi2	1.787	0.238	7.515	0.000	1.321	2.254	0.492
ant_pred1	~	lepi2	-1.350	0.370	-3.645	0.000	-2.076	-0.624	-0.388
ant_pred2	~	scoly1	-0.922	0.112	-8.217	0.000	-1.142	-0.702	-0.365
wasp1	~	scoly2	-1.138	0.258	-4.410	0.000	-1.643	-0.632	-0.321
ant_pred2	~	scoly2	1.867	0.337	5.539	0.000	1.206	2.527	0.508
ant_omni1	~	scoly2	-0.796	0.225	-3.538	0.000	-1.237	-0.355	-0.338
spider1	~	tree1	0.745	0.111	6.707	0.000	0.527	0.963	0.700
ant_pred2	~	tree1	-2.415	0.187	-12.920	0.000	-2.781	-2.048	-1.024
ant_omni1	~	tree1	-0.627	0.145	-4.312	0.000	-0.911	-0.342	-0.415
chilo1	~	tree1	0.640	0.161	3.980	0.000	0.325	0.955	0.456
scoly2	~	tree1	0.364	0.078	4.673	0.000	0.211	0.516	0.567
ant_omni1	~	tree2	0.742	0.208	3.572	0.000	0.335	1.149	0.363
wasp1	~	tree2	-1.202	0.226	-5.327	0.000	-1.644	-0.759	-0.391
ant_pred2	~	weevil1	0.246	0.070	3.492	0.000	0.108	0.384	0.207
wasp1	~	weevil2	0.865	0.103	8.420	0.000	0.663	1.066	0.439
ant_pred1	~	ceramby1	-0.458	0.141	-3.243	0.001	-0.735	-0.181	-0.302
wasp1	~	lepi2	-0.817	0.253	-3.233	0.001	-1.312	-0.322	-0.233
chilo1	~	weevil1	0.290	0.084	3.444	0.001	0.125	0.455	0.409
chilo1	~	ceramby1	-0.326	0.107	-3.029	0.002	-0.536	-0.115	-0.345
scoly1	~	herb1	0.220	0.072	3.060	0.002	0.079	0.361	0.478
chilo2	~	scoly1	0.527	0.168	3.147	0.002	0.199	0.856	0.507
weevil2	~	herb2	0.379	0.128	2.956	0.003	0.128	0.629	0.490
ant_pred1	~	weevil1	0.322	0.114	2.820	0.005	0.098	0.546	0.283
ceramby2	~	tree2	-0.651	0.235	-2.769	0.006	-1.111	-0.190	-0.420
para1	~	ant_pred1	-0.269	0.127	-2.110	0.035	-0.519	-0.019	-0.313
ant_pred1	~	tree1	0.485	0.230	2.110	0.035	0.034	0.936	0.215
chilo1	~	weevil2	0.285	0.142	2.008	0.045	0.007	0.564	0.235
chilo1	~	tree2	0.597	0.333	1.794	0.073	-0.055	1.249	0.314
lepi2	~	tree1	0.195	0.113	1.725	0.084	-0.026	0.417	0.301

chilo1	~	ceramby2	0.374	0.218	1.718	0.086	-0.053	0.800	0.304
para1	~	scoly1	0.597	0.357	1.670	0.095	-0.104	1.297	0.287
Environmental effects									
weevil2	~	envir1	0.040	0.011	3.533	0.000	0.018	0.062	0.509
ant_pred2	~	envir1	-0.088	0.013	-6.936	0.000	-0.112	-0.063	-0.545
tree2	~	envir1	-0.036	0.006	-5.904	0.000	-0.048	-0.024	-0.721
ceramby2	~	envir1	0.036	0.012	3.049	0.002	0.013	0.059	0.462
ant_omni1	~	envir1	-0.034	0.011	-3.003	0.003	-0.056	-0.012	-0.331
ant_pred1	~	envir1	-0.063	0.022	-2.904	0.004	-0.106	-0.021	-0.411
spider2	~	envir1	0.017	0.006	2.634	0.008	0.004	0.029	0.351
herb2	~	envir1	-0.034	0.014	-2.471	0.013	-0.062	-0.007	-0.339
weevil1	~	envir1	0.047	0.020	2.343	0.019	0.008	0.086	0.346
herb1	~	envir1	0.044	0.022	2.033	0.042	0.002	0.086	0.319
wasp1	~	envir2	-0.057	0.012	-4.591	0.000	-0.081	-0.032	-0.256
herb2	~	envir2	0.072	0.022	3.207	0.001	0.028	0.116	0.494
spider1	~	envir2	0.033	0.011	3.021	0.003	0.011	0.054	0.315
tree2	~	envir2	0.020	0.008	2.635	0.008	0.005	0.035	0.278
tree1	~	envir2	0.033	0.015	2.206	0.027	0.004	0.063	0.343
scoly1	~	envir2	-0.030	0.014	-2.150	0.032	-0.058	-0.003	-0.336
weevil2	~	envir2	0.029	0.017	1.681	0.093	-0.005	0.064	0.262
Variances and covariances									
ant_pred2	~~	ant_omni2	-0.010	0.003	-3.555	0.000	-0.016	-0.005	-0.380
wasp1	~~	ant_omni2	0.027	0.008	3.563	0.000	0.012	0.042	0.700
ant_omni1	~~	ant_omni1	0.009	0.002	3.755	0.000	0.004	0.014	0.183
ant_omni2	~~	ant_omni2	0.036	0.007	5.428	0.000	0.023	0.048	1.000
ant_pred1	~~	ant_pred1	0.062	0.016	3.749	0.000	0.030	0.094	0.552
ant_pred2	~~	ant_pred2	0.021	0.005	4.261	0.000	0.012	0.031	0.174
ceramby1	~~	ceramby1	0.049	0.013	3.674	0.000	0.023	0.075	1.000
ceramby2	~~	ceramby2	0.010	0.003	3.674	0.000	0.004	0.015	0.330
chilo1	~~	chilo1	0.015	0.004	3.674	0.000	0.007	0.023	0.349
chilo2	~~	chilo2	0.015	0.004	3.674	0.000	0.007	0.024	0.743
envir1	~~	envir1	4.747	1.292	3.674	0.000	2.215	7.279	1.000
envir2	~~	envir2	2.330	0.634	3.674	0.000	1.087	3.573	1.000
herb1	~~	herb1	0.081	0.020	4.028	0.000	0.042	0.121	0.898
herb2	~~	herb2	0.031	0.009	3.674	0.000	0.015	0.048	0.641
para1	~~	para1	0.048	0.013	3.674	0.000	0.022	0.073	0.579
scoly1	~~	scoly1	0.013	0.003	3.674	0.000	0.006	0.019	0.659
scoly2	~~	scoly2	0.006	0.002	3.811	0.000	0.003	0.009	0.678
spider1	~~	spider1	0.006	0.002	3.674	0.000	0.003	0.010	0.260
spider2	~~	spider2	0.004	0.001	3.800	0.000	0.002	0.007	0.405
tree1	~~	tree1	0.019	0.005	3.978	0.000	0.010	0.029	0.883
tree2	~~	tree2	0.005	0.001	3.674	0.000	0.002	0.007	0.403
wasp1	~~	wasp1	0.041	0.011	3.674	0.000	0.019	0.063	0.363
weevil1	~~	weevil1	0.076	0.021	3.674	0.000	0.036	0.117	0.881
weevil2	~~	weevil2	0.014	0.004	3.674	0.000	0.007	0.021	0.475
lepi2	~~	lepi2	0.008	0.002	3.674	0.000	0.004	0.013	0.910
spider2	~~	ant_omni2	-0.006	0.002	-3.434	0.001	-0.009	-0.003	-0.470

ant_pred2	~~	ant_omni1	0.007	0.002	3.199	0.001	0.003	0.011	0.485
ant_pred1	~~	ant_omni2	-0.023	0.007	-3.418	0.001	-0.036	-0.010	-0.489
spider2	~~	ant_pred1	0.011	0.004	2.962	0.003	0.004	0.018	0.662
tree1	~~	herb1	0.020	0.007	2.880	0.004	0.006	0.034	0.512
tree2	~~	herb1	-0.010	0.004	-2.723	0.006	-0.017	-0.003	-0.503
weevil1	~~	scoly2	-0.012	0.005	-2.598	0.009	-0.021	-0.003	-0.542
tree1	~~	herb2	-0.011	0.004	-2.567	0.010	-0.020	-0.003	-0.458
scoly2	~~	ceramby1	-0.006	0.003	-1.978	0.048	-0.012	0.000	-0.346
spider2	~~	ant_omni1	-0.001	0.001	-1.851	0.064	-0.003	0.000	-0.222
ant_pred1	~~	chilo2	-0.005	0.003	-1.595	0.111	-0.011	0.001	-0.163
lepi2	~~	ceramby2	0.003	0.002	1.567	0.117	-0.001	0.006	0.316

b) top-down

Left hand side	Operator	Right hand side	Estimate	SE	z	P	CI lower	CI upper	Standard. Est.
Taxon relations									
tree2	~	ant_omni1	0.230	0.058	3.981	0.000	0.117	0.343	0.515
herb2	~	ant_omni1	0.381	0.103	3.711	0.000	0.180	0.582	0.339
scoly2	~	ant_omni1	-0.268	0.051	-5.221	0.000	-0.369	-0.167	-0.694
herb1	~	ant_omni1	-0.757	0.152	-4.991	0.000	-1.054	-0.459	-0.550
scoly1	~	chilo2	0.608	0.145	4.191	0.000	0.324	0.893	0.656
tree1	~	lepi2	0.485	0.092	5.296	0.000	0.305	0.664	0.348
herb2	~	para1	-0.321	0.084	-3.823	0.000	-0.485	-0.156	-0.348
chilo2	~	para1	-0.266	0.072	-3.710	0.000	-0.407	-0.126	-0.517
herb1	~	scoly1	1.049	0.180	5.817	0.000	0.696	1.403	0.444
tree1	~	scoly2	0.946	0.169	5.595	0.000	0.615	1.278	0.551
herb2	~	spider1	-0.805	0.192	-4.202	0.000	-1.180	-0.429	-0.461
lepi2	~	spider1	0.466	0.087	5.376	0.000	0.296	0.637	0.629
tree1	~	spider1	0.381	0.103	3.712	0.000	0.180	0.583	0.369
herb2	~	wasp1	-0.350	0.095	-3.682	0.000	-0.536	-0.164	-0.364
tree1	~	weevil2	-0.293	0.058	-5.080	0.000	-0.406	-0.180	-0.331
ceramby1	~	ant_pred1	-0.408	0.117	-3.479	0.001	-0.637	-0.178	-0.579
tree1	~	ant_pred2	-0.180	0.054	-3.363	0.001	-0.285	-0.075	-0.319
tree2	~	ceramby2	-0.260	0.080	-3.236	0.001	-0.418	-0.103	-0.411
herb1	~	spider1	0.895	0.258	3.474	0.001	0.390	1.400	0.418
ceramby1	~	ant_pred2	-0.356	0.118	-3.010	0.003	-0.588	-0.124	-0.427
weevil1	~	chilo1	0.500	0.170	2.949	0.003	0.168	0.833	0.360
tree1	~	chilo1	0.140	0.046	3.017	0.003	0.049	0.230	0.191
tree2	~	wasp1	-0.130	0.043	-3.003	0.003	-0.214	-0.045	-0.340
lepi2	~	ant_pred1	-0.121	0.042	-2.859	0.004	-0.204	-0.038	-0.353
herb2	~	lepi2	0.651	0.228	2.860	0.004	0.205	1.097	0.276
lepi2	~	spider2	0.545	0.187	2.919	0.004	0.179	0.911	0.499
weevil2	~	wasp1	0.242	0.083	2.905	0.004	0.079	0.406	0.377
tree1	~	wasp1	0.126	0.045	2.781	0.005	0.037	0.214	0.221
herb1	~	ant_pred2	0.389	0.141	2.761	0.006	0.113	0.665	0.332
tree1	~	weevil1	0.092	0.034	2.722	0.006	0.026	0.158	0.175
herb1	~	scoly2	-1.187	0.453	-2.620	0.009	-2.075	-0.299	-0.333
ceramby1	~	chilo2	-0.530	0.210	-2.526	0.012	-0.941	-0.119	-0.340

ceramby1	~	wasp1	0.278	0.117	2.373	0.018	0.048	0.508	0.331
herb2	~	weevil2	0.382	0.162	2.351	0.019	0.063	0.700	0.255
scoly1	~	spider2	0.436	0.189	2.313	0.021	0.067	0.806	0.327
lepi2	~	para1	0.089	0.040	2.248	0.025	0.011	0.167	0.229
scoly1	~	para1	0.167	0.076	2.215	0.027	0.019	0.316	0.351
ceramby1	~	spider2	1.070	0.494	2.167	0.030	0.102	2.038	0.476
weevil1	~	ant_pred1	0.248	0.116	2.130	0.033	0.020	0.475	0.272
scoly2	~	wasp1	-0.086	0.041	-2.107	0.035	-0.166	-0.006	-0.259
tree2	~	spider1	0.159	0.077	2.056	0.040	0.007	0.311	0.230
herb2	~	scoly1	0.379	0.189	2.004	0.045	0.008	0.750	0.197
scoly2	~	ant_pred2	0.061	0.032	1.932	0.053	-0.001	0.123	0.186
herb2	~	ant_pred2	0.172	0.089	1.925	0.054	-0.003	0.347	0.180
weevil2	~	chilo2	0.281	0.149	1.890	0.059	-0.010	0.572	0.235
wasp1	~	para1	-0.164	0.088	-1.869	0.062	-0.335	0.008	-0.171
tree1	~	spider2	-0.183	0.105	-1.737	0.082	-0.389	0.023	-0.120
scoly2	~	para1	-0.049	0.028	-1.724	0.085	-0.105	0.007	-0.155
weevil2	~	chilo1	0.163	0.102	1.597	0.110	-0.037	0.362	0.197
scoly1	~	wasp1	0.111	0.071	1.556	0.120	-0.029	0.251	0.223
herb2	~	chilo2	-0.285	0.188	-1.516	0.129	-0.653	0.083	-0.159
Environmental effects									
spider2	~	envir1	0.037	0.006	6.658	0.000	0.026	0.048	0.788
ant_omni1	~	envir1	-0.068	0.016	-4.315	0.000	-0.099	-0.037	-0.639
ceramby2	~	envir1	0.056	0.010	5.900	0.000	0.038	0.075	0.750
spider1	~	envir2	0.043	0.011	3.823	0.000	0.021	0.065	0.438
weevil2	~	envir2	0.070	0.015	4.666	0.000	0.041	0.099	0.611
herb2	~	envir2	0.127	0.019	6.692	0.000	0.090	0.165	0.743
lepi2	~	envir2	-0.028	0.009	-3.288	0.001	-0.045	-0.011	-0.391
ant_pred1	~	envir1	0.068	0.023	2.991	0.003	0.023	0.112	0.456
lepi2	~	envir1	-0.026	0.009	-2.918	0.004	-0.043	-0.009	-0.510
ant_pred2	~	envir2	-0.079	0.027	-2.888	0.004	-0.132	-0.025	-0.439
ant_pred2	~	envir1	-0.054	0.019	-2.830	0.005	-0.091	-0.017	-0.430
ceramby1	~	envir1	-0.062	0.023	-2.666	0.008	-0.107	-0.016	-0.591
wasp1	~	envir1	0.049	0.019	2.604	0.009	0.012	0.087	0.395
chilo1	~	envir1	0.042	0.017	2.481	0.013	0.009	0.075	0.431
chilo2	~	envir2	-0.024	0.013	-1.847	0.065	-0.050	0.001	-0.252
spider1	~	envir1	0.018	0.011	1.756	0.079	-0.002	0.039	0.269
scoly2	~	envir1	-0.010	0.007	-1.456	0.145	-0.023	0.003	-0.238
Variances and covariances									
ant_omni1	~~	ant_omni1	0.032	0.008	3.796	0.000	0.016	0.049	0.592
ant_omni2	~~	ant_omni2	0.038	0.009	4.138	0.000	0.020	0.056	1.000
ant_pred1	~~	ant_pred1	0.083	0.022	3.834	0.000	0.041	0.126	0.792
ant_pred2	~~	ant_pred2	0.047	0.013	3.674	0.000	0.022	0.072	0.623
ceramby1	~~	ceramby1	0.025	0.007	3.674	0.000	0.011	0.038	0.471
ceramby2	~~	ceramby2	0.012	0.003	3.674	0.000	0.005	0.018	0.437
chilo1	~~	chilo1	0.037	0.010	3.674	0.000	0.017	0.056	0.814
chilo2	~~	chilo2	0.014	0.004	3.674	0.000	0.007	0.022	0.669
envir1	~~	envir1	4.747	1.292	3.674	0.000	2.215	7.279	1.000

envir2	~~	envir2	2.330	0.634	3.674	0.000	1.087	3.573	1.000
herb1	~~	herb1	0.032	0.009	3.674	0.000	0.015	0.050	0.315
herb2	~~	herb2	0.011	0.003	3.674	0.000	0.005	0.017	0.164
para1	~~	para1	0.081	0.022	3.674	0.000	0.038	0.124	1.000
scoly1	~~	scoly1	0.009	0.002	3.674	0.000	0.004	0.014	0.484
scoly2	~~	scoly2	0.004	0.001	3.960	0.000	0.002	0.006	0.477
spider1	~~	spider1	0.016	0.004	3.838	0.000	0.008	0.025	0.736
spider2	~~	spider2	0.004	0.001	3.736	0.000	0.002	0.006	0.379
tree1	~~	tree1	0.004	0.001	3.929	0.000	0.002	0.007	0.187
tree2	~~	tree2	0.003	0.001	3.674	0.000	0.001	0.005	0.276
wasp1	~~	wasp1	0.060	0.014	4.183	0.000	0.032	0.089	0.814
weevil1	~~	weevil1	0.066	0.017	3.791	0.000	0.032	0.100	0.758
weevil2	~~	weevil2	0.015	0.004	3.674	0.000	0.007	0.022	0.479
lepi2	~~	lepi2	0.006	0.002	3.963	0.000	0.003	0.009	0.509
tree1	~~	herb1	0.009	0.003	3.259	0.001	0.004	0.015	0.760
scoly2	~~	weevil1	-0.010	0.003	-3.030	0.002	-0.017	-0.004	-0.644
wasp1	~~	ant_omni1	0.025	0.008	2.968	0.003	0.009	0.042	0.570
wasp1	~~	ant_omni2	0.022	0.007	2.955	0.003	0.007	0.036	0.455
ant_pred1	~~	ant_omni2	-0.021	0.008	-2.640	0.008	-0.037	-0.005	-0.371
lepi2	~~	weevil1	0.010	0.004	2.538	0.011	0.002	0.017	0.474
wasp1	~~	chilo1	-0.022	0.009	-2.515	0.012	-0.040	-0.005	-0.476
lepi2	~~	scoly2	-0.002	0.001	-2.411	0.016	-0.004	0.000	-0.476
chilo2	~~	ant_omni2	0.008	0.003	2.360	0.018	0.001	0.015	0.350
herb2	~~	tree1	-0.002	0.001	-2.351	0.019	-0.004	0.000	-0.330
spider1	~~	chilo1	0.011	0.005	2.327	0.020	0.002	0.021	0.461
lepi2	~~	ceramby2	0.003	0.002	2.228	0.026	0.000	0.006	0.390
spider2	~~	ant_pred1	0.007	0.003	2.178	0.029	0.001	0.012	0.364
spider1	~~	ant_omni2	0.009	0.004	2.154	0.031	0.001	0.017	0.348
scoly2	~~	ceramby2	-0.002	0.001	-2.059	0.039	-0.004	0.000	-0.308
spider1	~~	ant_omni1	-0.008	0.004	-1.977	0.048	-0.017	0.000	-0.367
chilo2	~~	ant_pred1	-0.013	0.007	-1.964	0.050	-0.026	0.000	-0.377
lepi2	~~	weevil2	0.003	0.002	1.835	0.066	0.000	0.006	0.295
spider2	~~	ant_omni1	-0.003	0.002	-1.800	0.072	-0.006	0.000	-0.252
ceramby1	~~	weevil1	0.010	0.006	1.714	0.086	-0.001	0.021	0.240
weevil2	~~	scoly2	0.002	0.001	1.652	0.099	0.000	0.004	0.229
wasp1	~~	spider1	-0.010	0.006	-1.599	0.110	-0.023	0.002	-0.323
ant_omni1	~~	chilo1	-0.010	0.007	-1.534	0.125	-0.023	0.003	-0.297

Table S5. Structural equation model results for *belowground species richness patterns*. a) *bottom-up* control and b) *top-down* control (see Figure S2). For abbreviations see Table S3

**a) bottom-up**									
**Left hand side**	**Operator**	**Right hand side**	**Estimate**	**SE**	**z**	**P**	**CI lower**	**CI upper**	**Standard. Est.**
Taxon relations									
acido	~	amf	0.810	0.177	4.578	0.000	0.463	1.157	0.758
chloroflexi	~	amf	0.802	0.183	4.390	0.000	0.444	1.160	0.752
chloroflexi	~	decomp	-0.577	0.144	-4.019	0.000	-0.858	-0.296	-0.573
bacte	~	ecm	-0.408	0.109	-3.746	0.000	-0.622	-0.195	-0.416
chloroflexi	~	ecm	-0.325	0.112	-2.890	0.004	-0.545	-0.104	-0.320
bacte	~	patho	0.411	0.147	2.806	0.005	0.124	0.699	0.411
bacte	~	amf	0.393	0.154	2.551	0.011	0.091	0.694	0.380
ecm	~	tree	0.416	0.175	2.375	0.018	0.073	0.759	0.416
alpha	~	patho	0.413	0.175	2.353	0.019	0.069	0.757	0.404
acido	~	herb	-0.247	0.110	-2.238	0.025	-0.463	-0.031	-0.243
acido	~	sapro	-0.280	0.128	-2.192	0.028	-0.529	-0.030	-0.266
chloroflexi	~	tree	0.248	0.113	2.186	0.029	0.026	0.470	0.244
sapro	~	herb	-0.280	0.132	-2.127	0.033	-0.538	-0.022	-0.290
patho	~	tree	0.322	0.164	1.967	0.049	0.001	0.643	0.328
bacte	~	decomp	-0.278	0.142	-1.958	0.050	-0.557	0.000	-0.286
amf	~	tree	0.261	0.139	1.882	0.060	-0.011	0.533	0.275
decomp	~	tree	0.216	0.133	1.627	0.104	-0.044	0.477	0.215
alpha	~	herb	0.204	0.131	1.563	0.118	-0.052	0.461	0.204
Environmental effects									
decomp	~	envir1	0.612	0.133	4.604	0.000	0.351	0.873	0.608
amf	~	envir1	0.465	0.151	3.078	0.002	0.169	0.762	0.490
decomp	~	envir2	0.339	0.110	3.074	0.002	0.123	0.555	0.337
herb	~	envir2	-0.453	0.172	-2.639	0.008	-0.789	-0.117	-0.453
sapro	~	envir1	0.351	0.151	2.320	0.020	0.054	0.648	0.363
acido	~	envir1	-0.279	0.124	-2.258	0.024	-0.522	-0.037	-0.275
Variances and covariances									
acido	~~	acido	0.593	0.154	3.843	0.000	0.291	0.896	0.598
alpha	~~	alpha	0.770	0.210	3.674	0.000	0.359	1.181	0.795
amf	~~	amf	0.595	0.154	3.871	0.000	0.294	0.896	0.684
bacte	~~	bacte	0.577	0.150	3.839	0.000	0.282	0.872	0.622
chloroflexi	~~	chloroflexi	0.543	0.148	3.674	0.000	0.253	0.833	0.549
decomp	~~	decomp	0.460	0.125	3.674	0.000	0.214	0.705	0.471
ecm	~~	ecm	0.797	0.217	3.674	0.000	0.372	1.221	0.827
envir1	~~	envir1	0.963	0.262	3.674	0.000	0.449	1.477	1.000
envir2	~~	envir2	0.963	0.262	3.674	0.000	0.449	1.477	1.000
herb	~~	herb	0.766	0.208	3.674	0.000	0.357	1.174	0.795
patho	~~	patho	0.828	0.225	3.674	0.000	0.386	1.269	0.892
sapro	~~	sapro	0.708	0.186	3.806	0.000	0.343	1.072	0.785
tree	~~	tree	0.963	0.262	3.674	0.000	0.449	1.477	1.000
acido	~~	chloroflexi	0.399	0.130	3.077	0.002	0.145	0.652	0.702

alpha	~~	bacte	0.438	0.148	2.955	0.003	0.147	0.728	0.657
decomp	~~	amf	0.268	0.104	2.572	0.010	0.064	0.473	0.513
amf	~~	sapro	0.258	0.110	2.343	0.019	0.042	0.474	0.398
sapro	~~	patho	0.305	0.143	2.131	0.033	0.024	0.585	0.398
acido	~~	bacte	0.130	0.065	1.988	0.047	0.002	0.258	0.222

b) top-down

Left hand side	Operator	Right hand side	Estimate	SE	z	P	CI lower	CI upper	Standard. Est.
Taxon relations									
ecm	~	bacte	-0.766	0.200	-3.840	0.000	-1.157	-0.375	-0.716
amf	~	chloroflexi	0.485	0.123	3.928	0.000	0.243	0.727	0.457
tree	~	chloroflexi	0.511	0.141	3.621	0.000	0.234	0.787	0.414
herb	~	sapro	-0.664	0.150	-4.432	0.000	-0.958	-0.370	-0.574
tree	~	decomp	0.419	0.142	2.952	0.003	0.141	0.698	0.348
tree	~	bacte	-0.429	0.149	-2.883	0.004	-0.721	-0.137	-0.359
tree	~	sapro	-0.376	0.155	-2.418	0.016	-0.680	-0.071	-0.324
herb	~	chloroflexi	0.526	0.220	2.391	0.017	0.095	0.958	0.427
tree	~	ecm	0.330	0.139	2.376	0.017	0.058	0.602	0.295
ecm	~	acido	0.363	0.154	2.366	0.018	0.062	0.664	0.340
herb	~	amf	0.400	0.171	2.335	0.020	0.064	0.736	0.345
ecm	~	alpha	0.455	0.200	2.280	0.023	0.064	0.846	0.425
tree	~	patho	0.349	0.158	2.216	0.027	0.040	0.658	0.287
patho	~	alpha	0.292	0.143	2.046	0.041	0.012	0.572	0.297
herb	~	acido	-0.421	0.207	-2.033	0.042	-0.828	-0.015	-0.353
patho	~	chloroflexi	0.332	0.167	1.990	0.047	0.005	0.659	0.327
decomp	~	acido	0.217	0.109	1.980	0.048	0.002	0.431	0.219
herb	~	ecm	0.290	0.152	1.904	0.057	-0.008	0.588	0.260
sapro	~	chloroflexi	0.309	0.174	1.779	0.075	-0.031	0.649	0.290
Environmental effects									
amf	~	envir1	0.501	0.137	3.667	0.000	0.233	0.769	0.487
decomp	~	envir1	0.643	0.127	5.052	0.000	0.394	0.892	0.648
decomp	~	envir2	0.294	0.098	3.000	0.003	0.102	0.486	0.296
patho	~	envir2	0.437	0.162	2.699	0.007	0.120	0.755	0.444
sapro	~	envir1	0.393	0.148	2.650	0.008	0.102	0.684	0.381
sapro	~	envir2	0.414	0.169	2.456	0.014	0.084	0.744	0.401
chloroflexi	~	envir2	-0.250	0.130	-1.920	0.055	-0.505	0.005	-0.258
Variances and covariances									
acido	~~	acido	0.963	0.262	3.674	0.000	0.449	1.477	1.000
alpha	~~	alpha	0.963	0.262	3.674	0.000	0.449	1.477	1.000
amf	~~	amf	0.565	0.146	3.879	0.000	0.279	0.850	0.554
bacte	~~	bacte	0.963	0.262	3.674	0.000	0.449	1.477	1.000
chloroflexi	~~	chloroflexi	0.847	0.230	3.674	0.000	0.395	1.298	0.934
decomp	~~	decomp	0.421	0.115	3.674	0.000	0.196	0.646	0.444
ecm	~~	ecm	0.680	0.185	3.674	0.000	0.317	1.043	0.617
envir1	~~	envir1	0.963	0.262	3.674	0.000	0.449	1.477	1.000
envir2	~~	envir2	0.963	0.262	3.674	0.000	0.449	1.477	1.000
herb	~~	herb	0.567	0.154	3.674	0.000	0.265	0.869	0.412

patho	~~	patho	0.637	0.173	3.674	0.000	0.297	0.977	0.682
sapro	~~	sapro	0.689	0.188	3.674	0.000	0.322	1.057	0.670
tree	~~	tree	0.437	0.119	3.674	0.000	0.204	0.670	0.317
chloroflexi	~~	acido	0.625	0.211	2.957	0.003	0.211	1.039	0.692
alpha	~~	bacte	0.673	0.226	2.976	0.003	0.230	1.116	0.699
decomp	~~	amf	0.296	0.104	2.846	0.004	0.092	0.500	0.607
ecm	~~	amf	0.231	0.105	2.210	0.027	0.026	0.437	0.374
sapro	~~	patho	0.272	0.138	1.974	0.048	0.002	0.542	0.411

Table S6. Structural equation model results for *aboveground species richness patterns.* a) *bottom-up* control and b) *top-down* control (see Figure S3). For abbreviations see Table S4

a) bottom-up

Left hand side	Operator	Right hand side	Estimate	SE	z	P	CI lower	CI upper	Standard. Est.
Taxon relations									
para	~	ant_omni	0.355	0.153	2.319	0.020	0.055	0.656	0.343
chilo	~	ceramby	0.451	0.206	2.187	0.029	0.047	0.855	0.481
ant_pred	~	herb	-0.270	0.149	-1.811	0.070	-0.563	0.022	-0.254
ceramby	~	herb	-0.308	0.134	-2.294	0.022	-0.571	-0.045	-0.290
spider	~	lepi	0.393	0.123	3.184	0.001	0.151	0.635	0.358
para	~	scoly	-0.291	0.177	-1.644	0.100	-0.638	0.056	-0.281
wasp	~	scoly	-0.468	0.103	-4.557	0.000	-0.669	-0.267	-0.446
ant_pred	~	tree	0.445	0.149	2.979	0.003	0.152	0.737	0.418
wasp	~	tree	0.458	0.102	4.490	0.000	0.258	0.658	0.436
para	~	wasp	0.355	0.169	2.104	0.035	0.024	0.686	0.360
spider	~	weevil	0.377	0.127	2.978	0.003	0.129	0.626	0.334
wasp	~	weevil	0.567	0.105	5.377	0.000	0.360	0.774	0.526
Environmental effects									
spider	~	envir1	-0.712	0.123	-5.771	0.000	-0.954	-0.470	-0.648
ant_pred	~	envir1	-0.508	0.149	-3.402	0.001	-0.800	-0.215	-0.478
ant_omni	~	envir1	-0.376	0.178	-2.109	0.035	-0.726	-0.027	-0.376
chilo	~	envir1	-0.442	0.219	-2.016	0.044	-0.871	-0.012	-0.443
wasp	~	envir1	0.176	0.102	1.724	0.085	-0.024	0.376	0.168
ceramby	~	envir1	0.642	0.131	4.911	0.000	0.386	0.898	0.604
weevil	~	envir2	-0.282	0.159	-1.779	0.075	-0.593	0.029	-0.290
scoly	~	envir2	0.380	0.178	2.137	0.033	0.032	0.729	0.380
herb	~	envir2	-0.453	0.172	-2.639	0.008	-0.789	-0.117	-0.453
Variances and covariances									
ant_omni	~~	ant_omni	0.827	0.225	3.674	0.000	0.386	1.268	0.859
ant_pred	~~	ant_pred	0.579	0.158	3.674	0.000	0.270	0.888	0.532
ceramby	~~	ceramby	0.600	0.163	3.674	0.000	0.280	0.920	0.551
chilo	~~	chilo	0.793	0.216	3.674	0.000	0.370	1.217	0.830
envir1	~~	envir1	0.963	0.262	3.674	0.000	0.449	1.477	1.000
envir2	~~	envir2	0.963	0.262	3.674	0.000	0.449	1.477	1.000
herb	~~	herb	0.766	0.208	3.674	0.000	0.357	1.174	0.795
lepi	~~	lepi	0.963	0.262	3.674	0.000	0.449	1.477	1.000
para	~~	para	0.607	0.165	3.674	0.000	0.283	0.931	0.587
scoly	~~	scoly	0.824	0.224	3.674	0.000	0.384	1.263	0.855
spider	~~	spider	0.396	0.108	3.674	0.000	0.185	0.608	0.340
tree	~~	tree	0.963	0.262	3.674	0.000	0.449	1.477	1.000
wasp	~~	wasp	0.271	0.074	3.674	0.000	0.126	0.415	0.255
ceramby	~~	weevil	-0.361	0.153	-2.360	0.018	-0.661	-0.061	-0.510
weevil	~~	weevil	0.837	0.228	3.674	0.000	0.391	1.284	0.916

b) top-down

Left hand side	Operator	Right hand side	Estimate	SE	z	P	CI lower	CI upper	Standard. Est.

Taxon relations									
tree	~	scoly	0.487	0.133	3.663	0.000	0.226	0.748	0.481
tree	~	wasp	0.820	0.170	4.840	0.000	0.488	1.153	0.795
weevil	~	wasp	0.605	0.132	4.565	0.000	0.345	0.864	0.633
ceramby	~	wasp	-0.461	0.139	-3.323	0.001	-0.733	-0.189	-0.487
scoly	~	para	-0.477	0.153	-3.118	0.002	-0.778	-0.177	-0.454
wasp	~	para	0.490	0.161	3.036	0.002	0.174	0.806	0.475
lepi	~	wasp	-0.440	0.167	-2.635	0.008	-0.768	-0.113	-0.441
tree	~	weevil	-0.435	0.177	-2.450	0.014	-0.782	-0.087	-0.402
lepi	~	spider	0.508	0.212	2.395	0.017	0.092	0.924	0.494
tree	~	ant_pred	0.277	0.135	2.049	0.041	0.012	0.542	0.260
herb	~	lepi	0.286	0.154	1.853	0.064	-0.016	0.588	0.301
scoly	~	ant_omni	0.265	0.153	1.727	0.084	-0.036	0.565	0.252
herb	~	ceramby	-0.280	0.162	-1.728	0.084	-0.599	0.038	-0.281
Environmental effects									
spider	~	envir1	-0.644	0.147	-4.376	0.000	-0.933	-0.356	-0.644
ceramby	~	envir1	0.677	0.143	4.728	0.000	0.396	0.957	0.692
scoly	~	envir2	0.417	0.153	2.721	0.007	0.117	0.717	0.396
ant_pred	~	envir1	-0.419	0.175	-2.401	0.016	-0.762	-0.077	-0.419
herb	~	envir2	-0.379	0.157	-2.410	0.016	-0.688	-0.071	-0.388
wasp	~	envir1	0.349	0.161	2.166	0.030	0.033	0.666	0.339
ant_omni	~	envir1	-0.376	0.178	-2.109	0.035	-0.726	-0.027	-0.376
weevil	~	envir2	-0.281	0.137	-2.054	0.040	-0.548	-0.013	-0.285
lepi	~	envir1	0.358	0.220	1.625	0.104	-0.074	0.789	0.347
Variances and covariances									
ant_omni	~~	ant_omni	0.827	0.225	3.674	0.000	0.386	1.268	0.859
ant_pred	~~	ant_pred	0.794	0.216	3.674	0.000	0.370	1.217	0.824
ceramby	~~	ceramby	0.472	0.128	3.674	0.000	0.220	0.723	0.512
chilo	~~	chilo	0.963	0.262	3.674	0.000	0.449	1.477	1.000
envir1	~~	envir1	0.963	0.262	3.674	0.000	0.449	1.477	1.000
envir2	~~	envir2	0.963	0.262	3.674	0.000	0.449	1.477	1.000
herb	~~	herb	0.644	0.175	3.674	0.000	0.301	0.988	0.701
lepi	~~	lepi	0.685	0.186	3.674	0.000	0.320	1.050	0.671
para	~~	para	0.963	0.262	3.674	0.000	0.449	1.477	1.000
scoly	~~	scoly	0.610	0.166	3.674	0.000	0.284	0.935	0.573
spider	~~	spider	0.563	0.153	3.674	0.000	0.263	0.864	0.585
tree	~~	tree	0.466	0.127	3.674	0.000	0.217	0.715	0.427
wasp	~~	wasp	0.677	0.184	3.674	0.000	0.316	1.038	0.660
weevil	~~	weevil	0.485	0.132	3.674	0.000	0.226	0.744	0.519

Table S7. Structural equation model results for *belowground Shannon diversity patterns.* a) *bottom-up* control and b) *top-down* control (see Figure S2). For abbreviations see Table S3

**a) bottom-up**									
**Left hand side**	**Operator**	**Right hand side**	**Estimate**	**SE**	**z**	**P**	**CI lower**	**CI upper**	**Standard. Est.**
Taxon relations									
chloroflexi	~	amf	0.422	0.140	3.009	0.003	0.147	0.697	0.392
alpha	~	herb	0.376	0.155	2.426	0.015	0.072	0.680	0.387
amf	~	herb	0.378	0.161	2.351	0.019	0.063	0.693	0.389
chloroflexi	~	sapro	0.316	0.136	2.330	0.020	0.050	0.582	0.302
bacte	~	herb	0.401	0.175	2.290	0.022	0.058	0.744	0.400
alpha	~	ecm	-0.311	0.143	-2.174	0.030	-0.592	-0.031	-0.320
alpha	~	amf	0.330	0.157	2.104	0.035	0.023	0.638	0.330
ecm	~	herb	0.388	0.189	2.054	0.040	0.018	0.758	0.388
chloroflexi	~	tree	0.214	0.105	2.039	0.041	0.008	0.420	0.204
alpha	~	sapro	0.245	0.141	1.738	0.082	-0.031	0.520	0.252
acido	~	sapro	0.305	0.176	1.732	0.083	-0.040	0.651	0.307
bacte	~	ecm	-0.293	0.175	-1.677	0.094	-0.636	0.049	-0.293
bacte	~	sapro	0.255	0.168	1.520	0.129	-0.074	0.583	0.254
acido	~	amf	0.256	0.181	1.413	0.158	-0.099	0.611	0.250
Environmental effects									
sapro	~	envir1	0.623	0.150	4.142	0.000	0.328	0.918	0.623
decomp	~	envir1	0.659	0.145	4.559	0.000	0.376	0.943	0.659
chloroflexi	~	envir2	-0.452	0.106	-4.266	0.000	-0.660	-0.244	-0.431
patho	~	envir2	0.390	0.177	2.203	0.028	0.043	0.738	0.390
amf	~	envir1	0.324	0.161	2.018	0.044	0.009	0.639	0.334
herb	~	envir2	-0.349	0.180	-1.934	0.053	-0.702	0.005	-0.349
ecm	~	envir2	0.284	0.189	1.502	0.133	-0.087	0.654	0.284
Variances and covariances									
acido	~~	acido	0.772	0.210	3.674	0.000	0.360	1.184	0.811
alpha	~~	alpha	0.489	0.133	3.674	0.000	0.228	0.749	0.537
amf	~~	amf	0.672	0.183	3.674	0.000	0.314	1.030	0.738
bacte	~~	bacte	0.730	0.199	3.674	0.000	0.340	1.119	0.757
chloroflexi	~~	chloroflexi	0.457	0.124	3.674	0.000	0.213	0.701	0.433
decomp	~~	decomp	0.544	0.148	3.674	0.000	0.254	0.834	0.565
ecm	~~	ecm	0.814	0.222	3.674	0.000	0.380	1.249	0.846
envir1	~~	envir1	0.963	0.262	3.674	0.000	0.449	1.477	1.000
envir2	~~	envir2	0.963	0.262	3.674	0.000	0.449	1.477	1.000
herb	~~	herb	0.846	0.230	3.674	0.000	0.395	1.297	0.878
patho	~~	patho	0.816	0.222	3.674	0.000	0.381	1.252	0.848
sapro	~~	sapro	0.589	0.160	3.674	0.000	0.275	0.903	0.611
tree	~~	tree	0.963	0.262	3.674	0.000	0.449	1.477	1.000
acido	~~	chloroflexi	0.364	0.134	2.712	0.007	0.101	0.627	0.612
**b) top-down**									
**Left hand side**	**Operator**	**Right hand side**	**Estimate**	**SE**	**z**	**P**	**CI lower**	**CI upper**	**Standard. Est.**

Taxon relations									
herb	~	alpha	0.611	0.155	3.933	0.000	0.307	0.915	0.511
amf	~	chloroflexi	0.599	0.145	4.122	0.000	0.314	0.884	0.621
tree	~	chloroflexi	0.552	0.162	3.420	0.001	0.236	0.869	0.556
herb	~	ecm	0.410	0.139	2.943	0.003	0.137	0.684	0.349
herb	~	acido	-0.371	0.139	-2.660	0.008	-0.644	-0.098	-0.315
patho	~	bacte	0.405	0.162	2.497	0.013	0.087	0.723	0.365
tree	~	alpha	-0.332	0.158	-2.097	0.036	-0.643	-0.022	-0.310
herb	~	bacte	0.257	0.153	1.681	0.093	-0.042	0.556	0.218
patho	~	acido	-0.262	0.162	-1.616	0.106	-0.580	0.056	-0.236
decomp	~	bacte	0.224	0.138	1.618	0.106	-0.047	0.495	0.228
Environmental effects									
sapro	~	envir1	0.623	0.150	4.142	0.000	0.328	0.918	0.623
decomp	~	envir1	0.632	0.138	4.575	0.000	0.361	0.903	0.643
chloroflexi	~	envir2	-0.444	0.131	-3.385	0.001	-0.701	-0.187	-0.418
patho	~	envir2	0.536	0.162	3.303	0.001	0.218	0.854	0.483
tree	~	envir2	0.505	0.171	2.948	0.003	0.169	0.841	0.479
alpha	~	envir1	0.365	0.158	2.315	0.021	0.056	0.675	0.372
Variances and covariances									
acido	~~	acido	0.963	0.262	3.674	0.000	0.449	1.477	1.000
alpha	~~	alpha	0.800	0.218	3.674	0.000	0.373	1.227	0.862
amf	~~	amf	0.618	0.168	3.674	0.000	0.288	0.947	0.614
bacte	~~	bacte	0.963	0.262	3.674	0.000	0.449	1.477	1.000
chloroflexi	~~	chloroflexi	0.894	0.243	3.674	0.000	0.417	1.372	0.825
decomp	~~	decomp	0.497	0.135	3.674	0.000	0.232	0.762	0.534
ecm	~~	ecm	0.963	0.262	3.674	0.000	0.449	1.477	1.000
envir1	~~	envir1	0.963	0.262	3.674	0.000	0.449	1.477	1.000
envir2	~~	envir2	0.963	0.262	3.674	0.000	0.449	1.477	1.000
herb	~~	herb	0.505	0.138	3.674	0.000	0.236	0.775	0.380
patho	~~	patho	0.685	0.186	3.674	0.000	0.320	1.050	0.577
sapro	~~	sapro	0.589	0.160	3.674	0.000	0.275	0.903	0.611
tree	~~	tree	0.630	0.171	3.674	0.000	0.294	0.966	0.588
chloroflexi	~~	acido	0.657	0.219	3.001	0.003	0.228	1.085	0.707
alpha	~~	bacte	0.384	0.184	2.082	0.037	0.023	0.745	0.437

Table S8. Structural equation model results for *aboveground Shannon diversity patterns.* a) *bottom-up* control and b) *top-down* control (see Figure S3). For abbreviations see Table S4

a) bottom-up

Left hand side	Operator	Right hand side	Estimate	SE	z	P	CI lower	CI upper	Standard. Est.
Taxon relations									
chilo	~	ceramby	0.683	0.158	4.320	0.000	0.373	0.993	0.673
chilo	~	herb	0.398	0.120	3.307	0.001	0.162	0.634	0.386
spider	~	tree	0.448	0.154	2.912	0.004	0.146	0.749	0.455
spider	~	herb	-0.361	0.154	-2.351	0.019	-0.662	-0.060	-0.367
ant_omni	~	scoly	0.407	0.176	2.319	0.020	0.063	0.752	0.407
chilo	~	tree	-0.279	0.124	-2.252	0.024	-0.522	-0.036	-0.270
para	~	ant_pred	-0.359	0.174	-2.057	0.040	-0.701	-0.017	-0.355
ant_pred	~	ceramby	0.439	0.228	1.925	0.054	-0.008	0.886	0.447
chilo	~	scoly	0.200	0.120	1.658	0.097	-0.036	0.436	0.193
ceramby	~	tree	0.232	0.144	1.611	0.107	-0.050	0.515	0.228
wasp	~	lepi	-0.261	0.165	-1.584	0.113	-0.583	0.062	-0.266
wasp	~	ceramby	-0.325	0.210	-1.549	0.121	-0.737	0.086	-0.338
scoly	~	herb	0.295	0.192	1.531	0.126	-0.083	0.672	0.295
para	~	scoly	0.264	0.174	1.513	0.130	-0.078	0.605	0.261
Environmental effects									
chilo	~	envir1	-0.872	0.157	-5.565	0.000	-1.179	-0.565	-0.844
ceramby	~	envir1	0.649	0.144	4.501	0.000	0.366	0.931	0.637
weevil	~	envir2	-0.539	0.162	-3.326	0.001	-0.857	-0.221	-0.539
wasp	~	envir1	0.520	0.214	2.433	0.015	0.101	0.938	0.530
herb	~	envir2	-0.349	0.180	-1.934	0.053	-0.702	0.005	-0.349
ant_pred	~	envir1	-0.407	0.232	-1.755	0.079	-0.862	0.048	-0.408
scoly	~	envir2	0.317	0.192	1.646	0.100	-0.060	0.694	0.317
Variances and covariances									
ant_omni	~~	ant_omni	0.803	0.219	3.674	0.000	0.375	1.231	0.834
ant_pred	~~	ant_pred	0.832	0.226	3.674	0.000	0.388	1.275	0.866
ceramby	~~	ceramby	0.540	0.147	3.674	0.000	0.252	0.828	0.542
chilo	~~	chilo	0.364	0.099	3.674	0.000	0.170	0.559	0.355
envir1	~~	envir1	0.963	0.262	3.674	0.000	0.449	1.477	1.000
envir2	~~	envir2	0.963	0.262	3.674	0.000	0.449	1.477	1.000
herb	~~	herb	0.846	0.230	3.674	0.000	0.395	1.297	0.878
lepi	~~	lepi	0.963	0.262	3.674	0.000	0.449	1.477	1.000
para	~~	para	0.789	0.215	3.674	0.000	0.368	1.209	0.805
scoly	~~	scoly	0.846	0.230	3.674	0.000	0.394	1.297	0.878
spider	~~	spider	0.614	0.167	3.674	0.000	0.286	0.941	0.658
tree	~~	tree	0.963	0.262	3.674	0.000	0.449	1.477	1.000
wasp	~~	wasp	0.704	0.192	3.674	0.000	0.328	1.079	0.762
weevil	~~	weevil	0.683	0.186	3.674	0.000	0.319	1.047	0.709

b) top-down

Left hand side	Operator	Right hand side	Estimate	SE	z	P	CI lower	CI upper	Standard. Est.
Taxon relations									

ceramby	~	chilo	0.364	0.122	2.982	0.003	0.125	0.603	0.363
tree	~	spider	0.483	0.169	2.866	0.004	0.153	0.813	0.483
scoly	~	ant_omni	0.459	0.162	2.825	0.005	0.140	0.777	0.448
weevil	~	ant_omni	0.341	0.133	2.565	0.010	0.080	0.601	0.311
herb	~	spider	-0.335	0.165	-2.027	0.043	-0.659	-0.011	-0.347
herb	~	chilo	0.332	0.165	2.010	0.044	0.008	0.656	0.344
ceramby	~	ant_pred	0.226	0.117	1.936	0.053	-0.003	0.454	0.225
scoly	~	para	0.330	0.172	1.913	0.056	-0.008	0.667	0.322
lepi	~	wasp	-0.332	0.174	-1.907	0.057	-0.673	0.009	-0.328
weevil	~	ant_pred	0.261	0.140	1.872	0.061	-0.012	0.535	0.238
ant_pred	~	para	-0.335	0.181	-1.847	0.065	-0.690	0.020	-0.335
ceramby	~	wasp	-0.225	0.125	-1.805	0.071	-0.469	0.019	-0.224
weevil	~	chilo	-0.231	0.133	-1.739	0.082	-0.491	0.029	-0.211
weevil	~	para	0.241	0.140	1.728	0.084	-0.032	0.515	0.220
weevil	~	wasp	0.224	0.133	1.684	0.092	-0.037	0.485	0.204
lepi	~	ant_omni	-0.289	0.174	-1.663	0.096	-0.631	0.052	-0.286
scoly	~	ant_pred	0.279	0.172	1.619	0.105	-0.059	0.617	0.273
Environmental effects									
ceramby	~	envir1	0.839	0.130	6.466	0.000	0.585	1.093	0.836
weevil	~	envir2	-0.624	0.132	-4.743	0.000	-0.882	-0.366	-0.569
wasp	~	envir1	0.353	0.180	1.958	0.050	0.000	0.706	0.353
spider	~	envir1	-0.335	0.181	-1.847	0.065	-0.690	0.020	-0.335
ant_omni	~	envir1	0.308	0.183	1.685	0.092	-0.050	0.667	0.308
chilo	~	envir1	-0.296	0.184	-1.610	0.107	-0.656	0.064	-0.296
Variances and covariances									
ant_omni	~~	ant_omni	0.871	0.237	3.674	0.000	0.407	1.336	0.905
ant_pred	~~	ant_pred	0.855	0.233	3.674	0.000	0.399	1.311	0.888
ceramby	~~	ceramby	0.354	0.096	3.674	0.000	0.165	0.542	0.364
chilo	~~	chilo	0.879	0.239	3.674	0.000	0.410	1.347	0.912
envir1	~~	envir1	0.963	0.262	3.674	0.000	0.449	1.477	1.000
envir2	~~	envir2	0.963	0.262	3.674	0.000	0.449	1.477	1.000
herb	~~	herb	0.703	0.191	3.674	0.000	0.328	1.079	0.785
lepi	~~	lepi	0.778	0.212	3.674	0.000	0.363	1.194	0.790
para	~~	para	0.963	0.262	3.674	0.000	0.449	1.477	1.000
scoly	~~	scoly	0.685	0.187	3.674	0.000	0.320	1.051	0.680
spider	~~	spider	0.855	0.233	3.674	0.000	0.399	1.311	0.888
tree	~~	tree	0.738	0.201	3.674	0.000	0.344	1.132	0.767
wasp	~~	wasp	0.843	0.229	3.674	0.000	0.393	1.293	0.876
weevil	~~	weevil	0.450	0.123	3.674	0.000	0.210	0.691	0.389
